# Rock-inhabiting fungi: terminology, diversity, evolution and adaptation mechanisms

**DOI:** 10.1080/21501203.2021.2002452

**Published:** 2021-12-27

**Authors:** Bingjie Liu, Rong Fu, Bing Wu, Xingzhong Liu, Meichun Xiang

**Affiliations:** a State Key Laboratory of Mycology, Institute of Microbiology, Chinese Academy of Sciences, Beijing, China; b University of Chinese Academy of Sciences, Beijing, China; c Department of Microbiology, College of Life Science, Nankai University, Tianjin, China

**Keywords:** Fungi, rock-inhabiting fungi, lithophilic fungi, lithotolerant fungi, species diversity, adaption mechanisms

## Abstract

Rock-inhabiting fungi (RIF) constitute an ecological group associated with terrestrial rocks. This association is generally restricted to the persistent colonisation of rocks and peculiar morphological features based on melanisation and slow growth, which endow RIF with significance in eukaryotic biology, special status in ecology, and exotic potential in biotechnology. There is a need to achieve a better understanding of the hidden biodiversity, antistress biology, origin and convergent evolution of RIF, which will facilitate cultural relic preservation, exploitation of the biogeochemical cycle of rock elements and biotechnology applications. This review focuses on summarising the current knowledge of rock-inhabiting fungi, with particular reference to terminology, biodiversity and geographic distribution, origin and evolution, and stress adaptation mechanisms. We especially teased out the definition through summing up the terms related to rock-inhabting fungi, and also provided a checklist of rock-inhabiting fungal taxa recorded following updated classification schemes.

## 1. Introduction

Rock represents the most ancient and widespread terrestrial niche among various substrates or habitats for life on earth (Gorbushina et al. 2002; Beraldi-Campesi 2013). During the long-term evolutionary history of fungi, rock-inhabiting fungi (RIF) forming black microcolonies on the surface of rocks have evolved. Most of them grow slowly, produce melanin, and mainly undergo meristematic development or produce yeast-like forms (Gorbushina et al. 1993; Wollenzien et al. 1995; Chertov et al. 2004). Based on these characteristics, RIF are also named microcolonial fungi, meristematic fungi or black yeasts according to different perspectives of morphology or physiology (de Hoog and Hermanides-Nijhof 1977; Staley et al. 1982; Sterflinger 2006). Compared to lichens, which symbiotically live with photosynthetic microorganisms such as Cyanobacteria or algae to form conspicuous thalli on rock surfaces, RIF generally refer to heterotrophic free-living eukaryotic microorganisms (Palmer et al. 1990) and thus can hardly be observed effortlessly. Rock-inhabiting black fungi exhibit excellent performance in a broad range of extreme environments, from hot tropical deserts to semidry and humid Mediterranean coasts and McMurdo Antarctic dry valleys with multiple erratic stresses, such as solar radiation, desiccation and rehydration, and temperature fluctuations (Staley et al. 1982; Selbmann et al. 2005; Onofri et al. 2014).

Although the existence of heterotrophic microorganisms on rocks was previously reported (Gromov 1957), it is generally believed that meticulous academic studies of RIF began with Staley et al. (1982), who observed dark microcolonial structures on bare rock surfaces without algae or lichen by scanning electron microscopy (SEM) as well as high rates of physiological activity by respiration detection. Further confirmation was achieved with pure isolates of black fungi that could recolonize clean sterile marble within 3–6 months in the laboratory (Gorbushina et al. 1993). Subsequently, microscopic observation indicated limited diagnostic features of morphology based on their meristematic development or yeast-like form and a lack of ascosporic or conidial sporification (Palmer et al. 1987; Gorbushina et al. 1993; Sterflinger 2006; Gorbushina 2007). Due to the lack of morphological characteristics, reliable species delimitation of RIF did not become practicable by molecular phylogenetic analysis until the last decade of the 20^th^ century (Bruns et al. 1991). Many microcolonial black fungi have been isolated and identified as new species and even higher ranks in recent years (Egidi et al. 2014; Isola et al. 2016; Sun et al. 2020). The molecular phylogeny of these fungal strains indicated their unique affiliation within *Dothideomycetes, Eurotiomycetes* and *Arthoniomycetes* in *Ascomycota* (Gueidan et al. 2008; Ruibal et al. 2008, 2009; Egidi et al. 2014). Furthermore, the phylogenetic frame of RIF within *Dothideomycetes* based on multiple genes was proposed at the order or family level by Ruibal et al. (2009) and Egidi et al. (2014). With novel taxa discovered from rocks, physiological studies of RIF have also been conducted, mainly including utilization of carbon or nitrogen sources (Nai et al. 2013) and resistance to harsh stresses such as desiccation, radiation, acids, hypersaline and temperature fluctuations (Palmer et al. 1987; Sterflinger and Krumbein 1995; Sterflinger 1998a, 1998b; Onofri et al. 2007; Gorbushina et al. 2008; Zakharova et al. 2013). In addition, the origin of this ecological group and its evolutionary relationship with other lifestyles, such as plant pathogens, black yeasts and lichens, were also observed (Gueidan et al. 2008, 2011; Ruibal et al. 2009; Abdollahzadeh et al. 2020).

Despite the extremely slow growth and thick melanised cell wall of RIF (Isola et al. 2011), their adaptation mechanism to harsh niches has been investigated by morphological observation, physiological testing, and comparative genome, transcriptome and proteome analyses (Tesei et al. 2012; Zakharova et al. 2013, 2014; Coleine et al. 2017, 2020). Gene editing and RNA interference approaches to elucidate unique RIF genes have been successfully established (Noack-Schönmann, Bus, et al. 2014) and applied in the typical rock-inhabiting fungus *Knufia petricola* (Voigt et al. 2020). RIF richness in nature has been well documented, and characterization of its antistress biological characteristics, significance in exobiology, and ecological functions has made significant progress in recent decades. However, many terms related to the fungi on rocks are not well defined and correctly used. There are also no calculations how many rock-inhabiting fungi (RIF) have been reported worldwide. This paper reviews advances in describing RIF in the areas of morphology, physiology, taxonomy, ecology, evolutionary biology, genomics, molecular biology and biotechnological applications (Selbmann et al. 2015; Prenafeta-Boldú et al. 2019; Favero-Longo and Viles 2020; Vasileiou and Summerer 2020). In addition, terms related to RIF and a RIF checklist are especially provided.

## 2 Terminology

Fungi that colonise rocks suffer multiple stresses and have evolved adaptation traits to cope with the hard conditions in the niches they reside. Various terms have been applied to describe fungi on rocks in the literature with overlapping meanings, such as rock-inhabiting fungi, lithophilic fungi, microcolonial fungi, meristematic fungi, and black yeasts. Although each term emphasises different characteristics, the fungi can be classified into different groups according to their predominant morphological characteristics (Sterflinger 2006). To precisely use those terms and assign the fungi to relevant groups, we discussed and provided a comprehensive understanding of each term.

### 2.1. Rock-inhabiting fungi (RIF)

“Rock-inhabiting fungi” (RIF) is an extensively used term when exploring taxonomy, evolution, ecology, physiology and molecular mechanisms to emphasise the “inhabiting” trait on rocks (Sterflinger and Krumbein 1995; Wollenzien et al. 1995). RIF gives a broad sense of emphasising the “rock” habitats that fungi colonise. However, neither the temporary colonisation of some ubiquitous hyphomycetes nor dormant spore contamination without physiological activity are recognised as inhabitants (Wollenzien et al. 1995; Sterflinger et al. 2012). Indeed, the term “rock inhabiting microbiota”, including either fungi or bacteria as well as algae colonising rocks, has been used in earlier studies (Urzì et al. 1995). Another infrequent term, “rock dwelling fungi”, highlighting the “inhabiting” feature, is sometimes adopted: these fungi utilise rocks not as a source of organic or inorganic nutrients but rather as a dwelling for colonisation and propagation (Gorbushina and Krumbein 2000). In addition, some more popular terms, such as “stone/rock eating fungi”, are employed to visually describe their colonisation and corrosion of rock surfaces (Urzì et al. 2000; Kirtzel et al. 2017).

### 2.2. Lithobiontic fungi

The term “Lithobiontic fungi” is derived from “lithobiont” through the ancient Greek etyma “litho-”, meaning “rocks” and “biont”, referring to “one having a (specified) mode of life”. Lithobiontic fungi generally refers to either slow-growing black yeast or extensive mould that lives on or inside rocks (Heinen and Lauwers 1986; Caretta et al. 2006). This term is much less frequently used than RIF. Some related concepts are more accurate or convenient to convey, for example, epilithic, chasmolithic, and chasmoendolithic/endolithic fungi to describe fungi colonising the surface, gathering in fissures and cracks, and those penetrating actively into the interior of rocks forming tunnels, respectively (Bentis et al. 2000; Miura and Urabe 2017). In addition, “endolithic fungi”, along with another derived phrase, “cryptoendolithic fungi”, are commonly used terms in extreme biology, especially studies of cryptoendolithic communities in the McMurdo Dry Valleys, Antarctica, which is known as the location most closely resembling the Martian environment on Earth (Palmer and Friedmann 1988; Selbmann et al. 2005; Onofri et al. 2015; Coleine et al. 2018).

### 2.3. Microcolonial fungi (MCF)

“Microcolonial fungi” (MCF) were proposed by Staley et al. (1982) to refer to the colony appearance of the fungal assemblage residing on mineral substrates, mostly rock surfaces but also glass or metal, based on an ultrastructural examination of the microcolonial structures of black or brown stains on desert rocks. Generally, microcolonial fungi, especially those from rocks, are morphologically identified as having meristematic or yeast-like growth, with alterations of each other to a certain extent (Gorbushina et al. 1993; Wollenzien et al. 1995; Sterflinger 2006).

### 2.4. Meristematic fungi

The term “meristematic fungi” was introduced by de Hoog and Hermanides-Nijhof (1977) to refer to fungi that form aggregates of thick-walled, melanised cells enlarging and reproducing by isodiametrical division. Meristematic growth by isodiametric cellular expansion, which results in a minimal surface/volume ratio, facilitates survival under extreme temperatures and desiccation and economises energy requirements (Wollenzien et al. 1995).

### 2.5. Black yeast

“Black yeast” refers a group of fungi that are quite heterogeneous from taxonomic and phylogenetic perspectives but have common melanised cell walls and form daughter cells by yeast-like multilateral or polar budding (de Hoog and Hermanides-Nijhof 1977; Sterflinger 2006). Most black yeasts additionally exhibit mycelial growth and generate conidia from simple phialides. Some meristematic fungi can also be classified morphologically as black yeast and vice versa. Both forms have close phylogenetic relationships (Sterflinger et al. 1999). Either meristematic fungi or black yeasts describe partial microscopic traits of filamentous melanised fungi not only from rock surfaces but also from other ecological niches, such as soils, plants, animals (Sterflinger 2006) and epilithic lichens (Selbmann et al. 2013). In particular, some black yeasts are pathogenic to humans, causing chromoblastomycosis as well as phaeohyphomycosis (Moreno et al. 2018).

### 2.6. Melanized/black/dematiaceous fungi

Many fungi, not limited to RIF, produce black pigments, mainly melanin, in fungal cell walls to make their colonies melanised (Revankar 2007; Revankar and Sutton 2010). Therefore, “melanised fungi”, “black fungi” or “dematiaceous fungi” frequently appear in the literature on RIF. It is noteworthy that the term “melanised” is a more accurate description and is more frequently used, especially concerning the opposite word “non-melanised” of melanin-mutated cells (Dadachova et al. 2007), while “dematiaceous” has gradually become disused and is restricted to ubiquitous, mostly plant-associated hyphomycetous fungi (Revankar 2007).

### 2.7. Lithophilic/lithotolerant fungi

**Figure 1.**
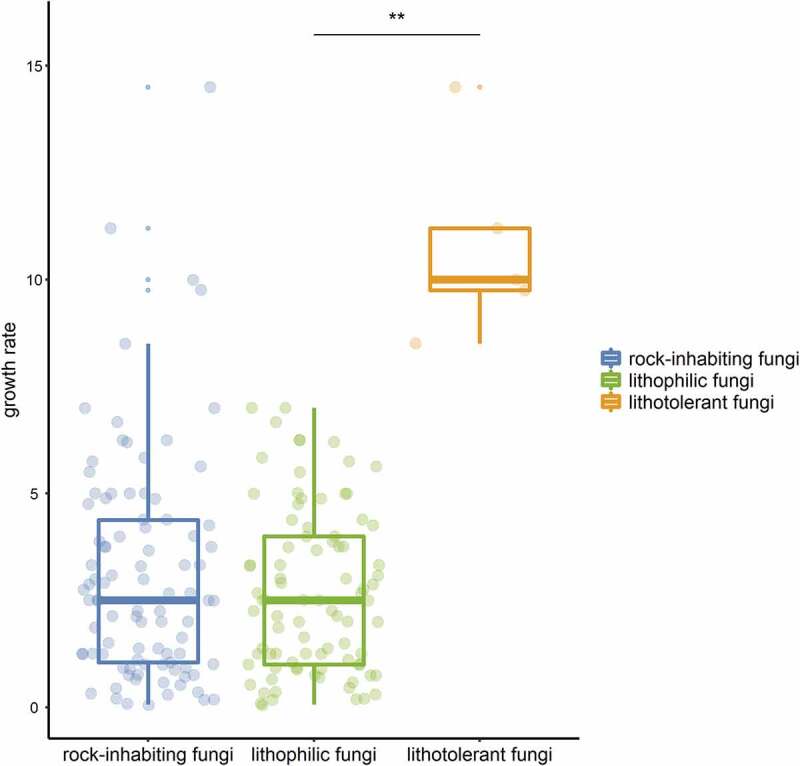
Growth rate comparison on culture plates between litholithic fungi and lithotolerant fungi of rock-inhabiting fungi. Values of plots represents growth rates (mm. per week) of published RIF species on culture plates. Significant difference between green and Orange plots represents the distinction between typical and nontypical RIF.

Many extremophilic fungi living in unique habitats suffering single stresses, such as high temperature and high salinity, can be separated into “-philic” and “-tolerant” fungi by some parameters, for instance, thermophilic fungi, which have a maximum temperature for growth at or above 50°C and a minimum temperature for growth at or above 20°C, while thermotolerant fungi have a minimum temperature for growth below 20°C (Cooney and Emerson 1964). These terms are well understood and widely accepted. Although the term “rock-inhabiting fungi” has been extensively accepted with its broad meaning, fungi on rocks often suffer multiple stresses and cannot be separated into “-phillic” and “-tolerant” by some parameters. Generally, fungal strains isolated from rocks are classified as typical RIF with a slow-growing meristematic or yeast-like microcolonial appearance or nontypical strains with a relatively fast-growing and melanised appearance (Wollenzien et al. 1995; Gorbushina and Krumbein 2000). Most typical RIF species are isolated only from rocks and are considered obligate rock dwellers, while nontypical RIF species can colonise various habitats and thus be facultative on rocks. Sometimes, certain nontypical RIF cannot survive the fluctuating stresses on rocks and might be regarded not as rock-inhabiting fungi but as contaminating fungi (Palmer et al. 1987; Wollenzien et al. 1995). The term “lithophile” refers to microorganisms that usually benefit from the rock niche either on the surface or within deep cracks by making use of light or mineral energy (Mikhailyuk 2008; Kuklinski 2009). Another term, “polyextremotolerant”, describes the tolerance and adaptation to multiple and changing stresses in rock habitats (Gostinčar et al. 2012, 2015; Grube et al. 2013). Therefore, the terms “lithophilic” and “lithotolerant” have been redefined and proposed to be applied to the distinction between typical and nontypical rock fungi. Lithophilic fungi grow extremely slow by less than 1 mm. per week in general, while relatively fast-growing lithotolerant fungi could reach to 1 mm. or more per week (Figure 1). Some distigushing parameters are also given (Table 1).

**Table 1.** Characteristics comparison between typical and nontypical rock fungi

Characteristics	lithophilic (typical) RIF		lithotolerant (nontypical) RIF	
	Niche	Culture	Niche	Culture
Habitats	rocks		rocks and other niches	
Growth	extremely slow	extremely slow	slow	moderate
Melanized cell wall	+	+	+	+
Microcolonial appearance	+	+	-	-
Thicken cell wall	+	+	±	±
Meristematic development	+	+	-	-
Black yeast form	±	±	±	±
Sporulation	-	-	-	±

## 3. Isolation of rock-inhabiting fungi

The methodology to isolate slow-growing melanised rock-inhabiting fungi from stone objects has been gradually improved. RIF were originally isolated by picking up dark microcolonies from rock surfaces onto MEA (malt extract agar) plates with a sterilised needle or scalpel under a dissecting microscope (Wollenzien et al. 1995; Sterflinger and Gorbushina 1997; Ruibal et al. 2018) or scraping the greyish-black patina of samples (de Leo et al. 2019) onto agar plates. Melanised fungal strains could also be obtained via the inoculation of disinfected rock samples on MEA plates (Selbmann et al. 2005). Disinfection of rock surfaces is necessary to reduce contamination by airborne spores or propagative fragments of some ubiquitous hyphomycetes (Su et al. 2015). Rock samples are generally disinfected by several rinses for several seconds with disinfectors such as 95% ethanol, 8% H_2_O_2_ solution or physiological saline containing 0.01% – 0.001% Tween 20 (Selbmann et al. 2005; Su et al. 2015) and finally washed with sterilised distiled water several times.

Recently, the pour plate method with ground rock pieces has been applied for RIF isolation, which has proven to be effective for large-scale rock samples (Ruibal et al. 2005). The isolation efficiency was also improved by the adoption of some special agar plates, such as 1/10 MEA (Su et al. 2015), DG18 (dichloran-glycerol 18% agar) or DRBC (dichloran-rose Bengal chlortetracycline), to provide oligotrophic conditions, low water activity or inhibition of fast-growing fungi (King Jr et al. 1979; Ruibal et al. 2008). Certainly, a lower culture temperature also avoids contamination by fast-growing fungi. Supplementation with stable antibiotics such as chloramphenicol, streptomycin sulphate and terramycin in agar plates to suppress bacterial growth is beneficial for RIF purification (King Jr et al. 1979; Su et al. 2015; Sun et al. 2019, 2020). When appearing on DRBC plates, black colonies should be transferred onto MEA or PDA (potato extract agar) plates (Ruibal et al. 2005) for purification. Inoculation conditions can be flexible according to the circumstances of the sampled rocks to achieve a better isolation effect, for example, an adjustment of the appropriate temperature ranging from 10 to 25 degrees (Ruibal et al. 2005; Hubka et al. 2014; Su et al. 2015; Sun et al. 2019; Sun et al. 2020), 12 h of fluorescent light exposure, and continual removal of rapidly extending unpigmented colonies (Ruibal et al. 2005).

## 4. Research progress

### 4.1. Biodiversity and geographic distribution

Phylogenetic analysis has provided an essential tool for RIF identification. Worldwide, RIF investigations have been carried out in the past few decades. Rock samples were collected in environments ranging from hot deserts in subtropical and tropical areas (Staley et al. 1982; Sterflinger et al. 2012) to the cold McMurdo Dry Valleys in the Antarctic (Selbmann et al. 2005, 2008; Egidi et al. 2014) or from moderately humid and semidry Mediterranean coasts (Wollenzien et al. 1995, 1997; Sterflinger et al. 1997; Onofri et al. 2014) to mountain peaks in the Andes, Alps and Indian Himalayan ranges (Egidi et al. 2014; Hubka et al. 2014; Selbmann et al. 2014; Su et al. 2015; Sun et al. 2020). Sampling was distributed in Europe, Asia, Antarctica and America, including at least 16 countries. The rock samples included natural field rocks such as granite, marble, pegmatite, quartz, limestone and sandstone and litholic heritages such as museums, cathedrals, temples, cemeteries and ancient caves, chambers of historical sites, roof tiles and metro systems (Sert et al. 2007a, 2007b, 2011, 2012; Sert and Sterflinger 2010; Martin-Sanchez et al. 2012; Egidi et al. 2014; Isola et al. 2016; Réblová et al. 2016; Kiyuna et al. 2018; Trovão et al. 2019).

Many melanised microcolonial fungal strains isolated on rocks from various extreme environments have raised great interest in the hidden species diversity of RIF worldwide. It was a formidable task to identify slow-growing fungi with yeast-like morphology, meristematic development, or extremely thin hyphal structures (Gorbushina et al. 1993; Ascaso et al. 1995; Wollenzien et al. 1995) until the development of molecular phylogenetic analysis and its application to fungal taxonomy (Sterflinger et al. 1997; Taylor et al. 2000).

**Figure 2.**
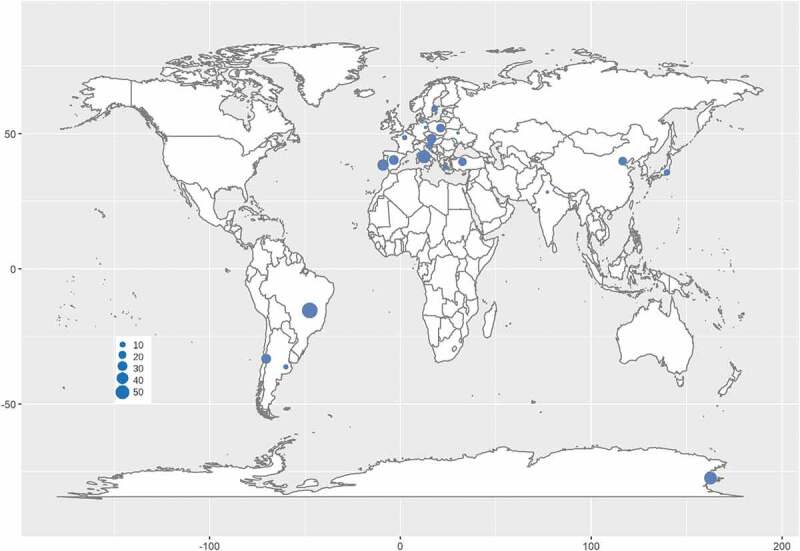
Distribution of rock-inhabiting fungi. Size of blue circles represents the genus number of rock-inhabiting fungi.

Although RIF are an ecological group of fungi, their phylogenetic positions are mainly affiliated with *Dothideomycetes* and *Eurotiomycetes* in Ascomycota (Gueidan et al. 2008; Ruibal et al. 2009; Egidi et al. 2014), as well as an unidentified lineage closely related to *Arthoniomycetes* (Ruibal et al. 2009). Furthermore, dothideomycetous RIF mainly cluster in the orders of *Capodiales* s. lat., *Dothideales* and *Myriangiales* in *Dothideomycetidae*, and *Coniosporiales, Pleosporales* and *Venturiales* in *Pleosporomycetidae* (Sterflinger et al. 1999; Ruibal et al. 2009; Egidi et al. 2014). Rencently, *Capnodiales* s. lat. was shown to be polyphyletic and separated into 7 orders, namely, *Capnodiales*. str., *Cladosporiales, Comminutisporales, Mycosphaerellales, Neophaeothecales, Phaeothecales* and *Racodiales* (Abdollahzadeh et al. 2020). Meanwhile, eurotiomycetous RIF are mostly gathered in *Chaeotothyriales* within *Chaeotothyriomycetidae* along with a few taxa in *Eurotiales* and *Verrucariales* (Sterflinger and Hain 1999; Gueidan et al. 2008). The RIF lineages closely related to A*rthoniomycetes* did not seem to form a monophyletic group, and their position in this class are not clear yet (Ruibal et al. 2009). According to the updated fungal nomenclature based on the databases of the Index Fungorum (http://www.indexfungorum.org) and MycoBank (http://www.mycobank.org) and a recent phylogenetic revision (Abdollahzadeh et al. 2020), more than 175 RIF species distributed in at least 16 countries worldwide are recorded in 1 phylum, 2 classes, 11 orders, 19 families and 64 genera, among which 1 new family, 27 new genera and 95 new species were established recently (Table 2; Figure 2).

**Table 2.** Species numbers and their affiliation of rock-inhabiting fungi

Phylum	Class	Subclass	Order	Family	Genus	Total RIF species	New species	Typical RIF	Nontypical RIF
*Ascomycota*	-	-	-	-	*Knufia*	9	9	9	0
	*Dothideomycetes*	-	-	-	*Cryomyces*	4	, 4	4	0
		-	-	-	*Rupestriomyces*	3	3	3	0
		-	-	-	*Saxomyces*	2	2	2	0
		-	-	-	*Spissiomyces*	3	2	3	0
		-	*Coniosporiales*	*Coniosporiaceae*	*Coniosporium*	3	3	3	0
		*Dothideomycetidae*	*Capnodiales*	-	*Arthrocatena*	1	1	1	0
				-	*Capnobotryella*	5	4	5	0
				-	*Catenulomyces*	1	1	1	0
				-	*Constantinomyces*	6	6	6	0
				-	*Elasticomyces*	1	1	1	0
				-	*Friedmanniomyces*	2	2	2	0
				-	*Hyphoconis*	1	1	1	0
				-	*Incertomyces*	2	2	2	0
				-	*Lapidomyces*	1	1	1	0
				-	*Meristemomyces*	1	1	1	0
				-	*Monticola*	1	1	1	0
				-	*Oleoguttula*	1	1	1	0
				-	*Penidiella*	1	0	1	0
				-	*Perusta*	1	1	1	0
				-	*Pseudotaeniolina*	1	1	1	0
				-	*Ramimonilia*	1	1	1	0
				-	*Recurvomyces*	1	0	1	0
				-	*Saxophila*	1	1	1	0
				-	*Vermiconidia*	4	4	4	0
				*Aeminiaceae*	*Aeminium*	1	1	1	0
				*Capnodiaceae*	*Leptoxyphium*	>1	0	0	1
				*Paradevriesiaceae*	*Paradevriesia*	1	1	1	0
				*Teratosphaeriaceae*	*Acrodontium*	>1	1	0	>1
					*Austroafricana*	>1	0	0	>1
					*Catenulostroma*	1	0	1	0
					*Hortaea*	2	0	2	0
					*Neocatenulostroma*	1	0	1	0
			*Cladosporiales*	*Cladosporiaceae*	*Cladosporium*	>10	0	0	>10
					*Rachicladosporium*	6	6	6	0
					*Verrucocladosporium*	>1	0	0	>1
			*Dothideales*	*Dothioraceae*	*Aureobasidium*	>1	0	0	>1
					*Endoconidioma*	>1	0	0	>1
					*Hormonema*	1	1	1	0
					*Pringsheimia*	>1	0	0	>1
			*Mycosphaerellales*	*Extremaceae*	*Extremus*	2	2	2	0
				*Mycosphaerellaceae*	*Pseudocercospora*	>1	0	0	>1
				*Neodevriesiaceae*	*Neodevriesia*	6	4	6	0
			*Neophaeothecales*	*Neophaeothecaceae*	*Neophaeotheca*	1	0	1	0
			*Phaeothecales*	*Phaeothecaceae*	*Phaeotheca*	>1	0	0	>1
		*Pleosporomycetidae*	*Pleosporales*	-	*Phoma*	>5	0	0	>5
				*Periconiaceae*	*Periconia*	>5	0	0	>5
				*Pleosporaceae*	*Alternaria*	>5	0	0	>5
			*Venturiales*	*Sympoventuriaceae*	*Ochroconis*	2	2	0	2
	*Eurotiomycetes*	*Eurotiomycetidae*	-	-	*Sarcinomyces*	1	1	1	0
			*Chaetothyriales*	-	*Bacillicladium*	1	1	1	0
				-	*Bradymyces*	4	4	4	0
				-	*Neophaeococcomyces*	>1	0	0	>1
				*Cyphellophoraceae*	*Cyphellophora*	2	2	2	0
				*Herpotrichiellaceae*	*Cladophialophora*	5	4	5	0
					*Exophiala*	6	5	6	0
					*Phaeococcomyces*	1	0	1	0
					*Phialophora*	1	0	1	0
					*Rhinocladiella*	1	0	1	0
				*Trichomeriaceae*	*Anthracina*	1	1	1	0
					*Lithohypha*	2	2	2	0
					*Trichomerium*	4	4	4	0
			*Eurotiales*	*Aspergillaceae*	*Aspergillus*	>10	0	0	>10
					*Penicillium*	>10	0	0	>10
1	2	3	11	19	64	>175	95	108	>65

*Family and genus names in bold are novel taxa of rock-inhabiting fungi

RIF have been deemed a typical ecological group rather than a phylogenetic lineage, as they were revealed to be polyphyletic based on either teleomorphic traits or molecular data (Golubic et al. 1981; Wollenzien et al. 1995; Sterflinger et al. 1999). These lineages possess various lifestyles, such as plant pathogens, epiphytes, saprobes and lichen-forming fungi in *Dothideomycetes* (Ruibal et al. 2009) and aquatic, ant-associated, myco-parasitic and human opportunistic lifestyles in *Chaetothyriales* (Teixeira et al. 2017). RIF often form early phylogenetic clades in *Dothideomycetes* and *Eurotiomycetes*, triggering the question of whether the rock surface was a terroir for ancient lineages or a reservoir for plant-associated fungi (Ruibal et al. 2009).

A phylogenomic approach was adopted to resolve relationships among fungi in *Dothideomycetes*, and two rock-inhabiting fungal genera, *Lichenothelia* and *Saxomyces*, have been suggested to be early diverging lineages. Ancestral character state reconstruction also suggested that the rock-inhabiting lifestyle is ancestral within the class (Ametrano et al. 2019). Another ancestral state reconstruction of *Capnodiales* s. lat., the second largest order possessing the most RIF taxa within *Dothideomycetes*, revealed its saprobic lifestyle, not specifically referring to its extremotolerant rock-inhabiting but rather an ancestral state relative to parasitic, epi- or ecto-phytic and lichenized lifestyles (Abdollahzadeh et al. 2020). Reconstructions of RIF-related orders within *Eurotiomycetes* also revealed that the most recent common ancestor of *Verrucariales* and *Chaetothyriales* is a nonlichenized rock inhabitant. *Verrucariales* is one of the independent groups where lichenization has evolved on a hostile rock surface that might have favoured the shift to a symbiotic lifestyle (Gueidan et al. 2008). The origin of RIF has been estimated back to the evolution of *Dothideomycetes* and *Chaetothyriales* in the Late Devonian and a much later period, the Middle Triassic, respectively, through a relaxed clock model combined with several fossil and secondary calibrations, which is confirmed by the fact that the lineages of RIF related to *Chaetothyriales* show a much narrower phylogenetic spectrum than *Dothideomycetetes*. The Devonian and Triassic epochs share characteristics of large arid landmasses, but the temperatures of the latter were much cooler than those of the former (Gueidan et al. 2011).

The evolutionary footprint was also traced by the shared characteristics between rock inhabiting fungi and other lifestyles within *Dothideomycetes* or *Chaetothyriales*. Melanised fungi form microcolonies not only on mineral substrates such as rocks but also on various extensive hard material surfaces, including outdoor and indoor glass, plastics, roof tiles, solar panels, moisteners, humidifiers and dishwashers (Gostinčar et al. 2011; Ruibal et al. 2018). Indeed, the bounds of this special group can be expanded to be polyextremotolerant, since stress factors on rocks are partly found in hyperhaline, acidic and radiation- or toxin-polluted soils/waters, where many rock dwellers have been observed to be holotolerants, acidotolerants and exotic carbohydrate degraders (Selbmann et al. 2008; Isola et al. 2013; Coleine et al. 2020; Su et al. 2020; Tesei et al. 2020). Although the CFPP-like (carbon fixation pathway of plants) pathway from fungi for carbon fixation is incomplete due to the absence of the unique enzymes Rubisco (ribulose-1,5-bisphosphate carboxylase-oxygenase) and PRK (phosphoribulokinase) (Lyu et al. 2015), nutritional pathways, especially in RIF, might have developed, since lithotrophs or photoheterotrophs were reported among hypersaline-tolerant and endolithic marine fungi through the oxidation of elemental sulfur, iron and manganese or light-driven rhodopsin transmembrane protons and sodium pumps for extra energy (Gleason et al. 2019) and radiotropism in reactor cooling water through melanin-dominant radiation adaptation, capture and energy transduction (Dadachova and Casadevall 2008). Equal photoreceptors (PRs) were detected in RIF genomes, which are similar to the plant pathogen *Botrytis cinerea* (*Leotiomycetes*), in which PRs function in sensing and avoiding sunlight stresses and locating susceptible hosts (Schumacher and Gorbushina 2020). Co-culture experiments confirmed the capacity for RIF to maintain structural and functional associations, an underlying protolichen format, with bacteria, especially cyanobacteria (Gorbushina et al. 2005; Gueidan et al. 2008; Ruibal et al. 2009; Gostinčar et al. 2012). Some studies also indicated that ant‐associated lifestyles might have driven the diversification of *Chaetothyriales* due to the metabolic capabilities of toxic compounds produced by ants (Teixeira et al. 2017; Moreno et al. 2018). Regarding the pathogenicity presented by rock dwellers, there was a statistically significant co-occurrence in the orders of *Capnodiales, Dothideales*, and *Chaetothyriales* in a kingdom-wide phylogenetic analysis (Gostinčar et al. 2018b). Although physiological virulence characteristics such as tolerance to raised temperature in warm-blooded animals or haemolysis on blood agar were rarely significantly detected in typical melanized rock fungi (Gostinčar et al. 2011; Gonçalves et al. 2017), virulence-associated genes were shown to be unnecessary in opportunistic human fungi (Gostinčar et al. 2018b). As melanin has exhibited great talent for oxidative stress resistance, virulence, camouflage, immune defence and copper and zinc homeostasis in mammalian tissues (Cunha et al. 2010; Silva-Bailao et al. 2018; Freitas et al. 2019; Smith and Casadevall 2019), it might be an evolutionary alternative form of stress adaptability to human opportunistic pathogenicity (Gostinčar et al. 2018b).

There are several hypotheses to elucidate the evolutionary dynamics of rock-inhabiting fungi. A proposal highlighting the potential oneness between rock-inhabiting fungi and their related lifestyles within both *Dothideomycetes* and *Chaetothyriales* has been mentioned. Some niches, such as rock or hard material surfaces, phyllosphere, and living mammalian tissues, which are widely divergent as described, share some main ecological similarities, such as increased temperature, osmotic stress, UV radiation and oxygenic action (Sterflinger 2006). Another proposal may underlie the separate evolutionary tactics of the two classes. Lineages with true extremophilic traits, such as psychrophiles, acidophiles or halophiles, tend to cluster in *Dothideomycetes*, while those with a larger spectrum of assimilative abilities, such as thermotolerance, toxin degradation or opportunistic pathogenicity, cluster in *Chaetothyriales* (Isola et al. 2016).

### 4.2. Niche adaptation mechanisms

#### 4.2.1 Morphological and physiological adaptation traits

Adaptative evolution of RIF to a niche with multiple stresses has resulted in their idiosyncratic cellular structures. One is the strong accumulation of melanin in the cell walls, which is thickened with multilayered construction, and the other is microcolonies forming on solid substrates via meristematic or yeast-like growth.

Melanisation endows RIF with inimitable survival advantages compared to soil- or plant-related fungi (Ruibal et al. 2008), algae and even lichens (Perry et al. 2004; Scalzi et al. 2012; Pacelli et al. 2017b) among eukaryotic organism domains. Fungal melanin is regarded as a strong safeguard against a wide range of electromagnetic radiation, from nonionising UV radiation to even ionising X-rays, gamma radiation beta-radiation and deuterons (Casadevall et al. 2017; Vasileiou and Summerer 2020). In addition, melanin exhibits sufficient protection for fungal cell growth under other stresses by performing oxidative or free radical scavenging, withstanding dehydration or high temperatures, and increasing mechanical-chemical cellular strength and heavy metal ion binding (Cordero and Casadevall 2017). Other protective soluble compounds with multiple stress resistance to desiccation, temperature, and irradiation also tend to be accumulated in black fungal cells. Intracellular mycosporines, such as mycosporine-glutaminol, mycosporine-glutaminol-glucoside, mycosporine-glutamicol, and mycosporine-glutamicol-glucoside, act as UV filters, antioxidants and minor osmolytes (Volkmann et al. 2003; Gorbushina et al. 2008) as well as possible hyphal development regulators of nonexpansive intracolonial growth (Gorbushina 2003). Diverse carotenoids including carotene, didehydrolycopene, lycopene, phytoene, torulene and torularhodin contribute to cell membrane stabilisation (Gorbushina et al. 2008) as well as antioxidative function and cold resistance (Gorbushina 2003; Flieger et al. 2018). Accumulation of some regular modulating substances, such as trehalose, mannitol and glycerol (Sterflinger 1998a, 1998b; Coleine et al. 2021), assists in survival under high temperatures, desiccation and osmotic stress on rocks.

Additionally, extracellular polymeric substances (EPS) composed of polysaccharides, glycoproteins and enzymes around fungal cells form a complex network to survive against various stresses, including temperature fluctuations, low water availability, UV radiation, and nutrient deficiency (Gorbushina 2003; Omelon 2008; Noack-Schönmann et al. 2014b; Breitenbach et al. 2018). These EPSs are also involved in the interaction of RIF with algae, cyanobacteria and heterotrophic bacteria (Gorbushina and Broughton 2009)

Compared with the typical filamentous growth and spore spreading habits of normal fungi, RIF meristematic or yeast-like growth is the common format to survive in rock niches. Meanwhile, sexual reproduction was spontaneously abandoned, and typical conidiophores rarely develop for RIF (Sterflinger 2006; Gorbushina 2007). Along with the fluctuation of environmental conditions, drastical morphological alterations were observed from meristematic growth to yeast-like cells, even to merely thin hyphae (Wollenzien et al. 1995; Ruibal et al. 2008; de Leo et al. 2019). There are numerous benefits for RIF derived from this slow radial extension and extraordinary propagation:

1. Meristematic or yeast-like growth results in a microcolony that can be embedded in extracellular polymeric substances to have a thermodynamically optimal surface and efficient exchange process rate to protect against extensive evaporation (Gorbushina and Krumbein 2000; Gorbushina 2003);
2. Meristematic swelling of cells and the formation of endocondia also have advantages for water-independent propagation, such as budding or germination. (Wollenzien et al. 1995; Gorbushina 2003)
3. Morphological alteration from spherical yeast cells to the filamentous hyphal form responding to environmental fluctuation represents the flexible balance between adversity survival and nutritional exploration (Chertov et al. 2004; Tonon et al. 2021).

#### 4.2.2. Genomic and proteomic features

RIF adapted to the rock niche have evolved special phenotypic traits, including morphological and physilogical characteristics and antistress biology. To understand the phenotypic traits, several typical RIF have been genome sequenced, including *Cryomyces antarcticus* MA 5682 (Sterflinger et al. 2014), *Friedmanniomyces endolithicu*s CCFEE 5311, *Friedmanniomyces simplex* CCFEE 5184, *Hortaea thailandica* CCFEE 6315 (Coleine et al. 2020), *Rachicladosporium antarcticum* CCFEE 5527 and *Rachicladosporium* sp. CCFEE 5018 (Coleine et al. 2017) from the coldest and most hyperarid desert McMurdo Dry Valleys of the Antarctic and one strain, *Coniosporium apollinis* CBS 100218 (Sterflinger et al. 2014), from the environment incorporating high temperature, desiccation and radiation occurring in the semiarid Mediterranean. Other melanized fungal strains isolated from hypersaline or peracid substrates or as human opportunistics were also genome sequenced (Table 3). Two strains, *C. antarcticus* MA 5682 and *F. endolithicus* CCFEE 5311, possessing excellent stress resistance against solar radiation, radioactivity, desiccation and oligotrophic conditions, were analysed in detail. The preliminary genome analysis of *C. antarcticus* MA 5682 indicated that there were no significant differences compared with the model filamentous fungus *Neurospora crassa* or other RIF strains, concerning either their genomic size (24 Mb) or GC content (53.84%) and the percentage (0.33) of repetitive sequences (Sterflinger et al. 2014). However, genome assembly and annotation of *F. endolithicus* CCFEE 5311 and *R. antarcticum* CCFEE 5527 showed that their genome sizes were double the average in black fungi with raletively high GC content (49–56.5%) (Coleine et al. 2017, 2020). Additionally, some genomic features unique to *Friedmanniomyces* spp. strains were detected, such as responses to X-ray radiation, DNA damage, and salt tolerance stress. It is noteworthy that the large genome size of *F. endolithicus* CCFEE 5311 (Coleine et al. 2020), similar to the halophilic strain *H. werneckii* EXF-2000 from hypersaline environments, suggests a large-scale genome duplication in the Antarctic species to adapt and survive in the hostile conditions of the ice-free areas of the Antarctic, which are prohibitive for most life forms (Lenassi et al. 2013). Another study proposed hybridization between two haploids in the genome of *H. werneckii*, other than endoreduplication, as suggested previously (Gostinčar et al. 2018a), which might provide novel ideas regarding the genomic features of RIF.

**Table 3.** Genomes of rock-inhabiting fungi and related strains

Species	Strain	Genome size (Mb)	GC (%)	Repeat (%)	tRNA	Gene	Location/Substrate	Reference
*Coniosporium apollinis*	CBS 100218	28.51	52.13	28	-	11,886	Microcolonial fungi, mediterranean region	Sterflinger et al. 2014
*Cryomyces antarcticus*	MA 5682	24.32	53.84	33	-	10,731	Cryptoendolithic fungi, Antarctica	Sterflinger et al. 2014
*Friedmanniomyces endolithicus*	CCFEE 5311	46.75	56.5	-	43	18,070	Cryptoendolithic fungi, Antarctica	Coleine et al. 2020
*Friedmanniomyces simplex*	CCFEE 5184	37.79	56.6	-	22	13,788	Cryptoendolithic fungi, Antarctica	Coleine et al. 2020
*Hortaea thailandica*	CCFEE 6315	23.89	55.5	-	23	8,801	Cryptoendolithic fungi, Antarctica	Coleine et al. 2020
*Rachicladosporium antarcticum*	CCFEE 5527	47.4	-	-	-	18,781	Cryptoendolithic fungi, Antarctica	Coleine et al. 2017
*Rachicladosporium sp.*	CCFEE 5018	-	-	-	-	18,892	Cryptoendolithic fungi, Antarctica	Coleine et al. 2017
*Acidomyces acidophilus*	BFW	21.87	54.8	-	41	10,549	Richmond Mine, California	Coleine et al. 2020
*Baudoinia panamericana*	UAMH 10762	29.88	49.5	-	-	-	Ethanol vapor	Coleine et al. 2020
*Cladosporium sphaerospermum*	UM 843	26.13	55.87	29	-	16,622	ubiquitous hyphomycte	Sterflinger et al. 2014
*Exophiala dermatitidis*	NIH/UT8656	26.37	51.51	33	-	10,020	opportunistics	Sterflinger et al. 2014
*Hortaea werneckii*	EXF-2000	49.89	53.5	-	28	15,649	Marine solar salterns, Slovenia	Coleine et al. 2020
*Hortaea werneckii*	EXF-2000	51.62	53.58	27	-	26,313	hypersaline	Sterflinger et al. 2014

*Species name in bold are typical rock-inhabiting fungi

The dynamic changes in whole-cell protein patterns of extremotolerant RIF under stresses also implied their peculiar potential adaptation mechanisms. The protein profiles of three niche-adapted groups of RIF were determined, e.g. *F. endolithicus* CCFEE 5208 (Tesei et al. 2012) and *C. antarcticus* MA 5682 (Zakharova et al. 2013, 2014), which are extremophilic cryptoendolithic fungi from cold and dry Antarctica; *Knufia perforans* (=*Coniosporium perforans*) MA 1299 (Tesei et al. 2012; Zakharova et al. 2013, 2014), which is a mesophilic but highly stress-tolerant microcolonial fungus from hot and dry environments in the Mediterranean; and *Exophiala jeanselmei* MA 2853 (Tesei et al. 2012; Zakharova et al. 2013, 2014), which is a rock-inhabiting black yeast closely related to opportunistic pathogens of humans. The 2-D protein spectra were not the same for the three types of RIF after desiccation stress, and both mesophilic strains *E. jeanselmei* and *K. perforans* (=*C. perforan*) showed clear production of small proteins (Zakharova et al. 2013). When RIF were stimulated with high temperature up to 40°C, the extremotolerant *C. antarcticu* did not show any response to desiccation but seemed to downregulate its metabolism. Nevertheless, compared with the mesophilic hyphomycete *Penicillium chrysogenum*, which expressed a higher number of proteins exhibiting real signs of temperature-induced reactions (Tesei et al. 2012), all three groups of RIF decreased their expressed protein numbers, indicating a downregulation of their metabolism under stress (Tesei et al. 2012). When exposed to thermophysical Mars-like conditions in terms of simulant gas composition, pressure and humidity with day-night fluctuating simulations of radiation spectra ranging from 200 nm to 2200 nm and temperatures ranging from −55°C to 15°C, those fungi showed certain stable survival traits, with upregulation of some unidentified proteins, significant decreases in protein numbers detected and no expression of any additional proteins such as heat shock proteins (HSPs) (Zakharova et al. 2014). Indeed, it would be a better survival strategy to express a specialized basic set of proteins in RIF compared with the production of HSPs, which is more energy-consuming (Naranjo‐Ortiz and Gabaldón 2019). The “Shed light in The daRk lineagES of the fungal tree of life” (STRES) project focusing on extremotolerant black fungi in different ecologies and life-styles, e.g. black yeasts, ant- and lichen-associated fungi, rock-inhabiting fungi etc., by genome sequencing and analysis coupled with transcriptomics and metabolomics experiments, may provide a comprehensive understanding of RIF (Selbmann et al. 2020).

#### 4.2.3. Metabolic response to stresses

The metabolic adaptation of RIF to stresses has been investigated in recent years. *C. antarcticu*, a typical melanized RIF, constitutively synthesizes melanin pigments by both the 1,8-dihydroxynaphthalene (DHN) and 1,3–4 dihydroxyphenylalanine (L-DOPA) pathways (Pacelli et al. 2020). The resistance of melanized fungi to cosmic and terrestrial ionizing radiation suggests that melanin plays a pivotal role in radioprotection. Melanin afforded protection for both *Cryptococcus neoformans*, a fast-growing pathogenic basiodiomycete, and *C. antarcticu* against high-dose deuterons, as well as *C. antarcticus* against X-rays. Deuterons increased XTT activity in melanized cells of these two species, which reflected the metabolic activity of the cells (Pacelli et al. 2017a). A similar response was observed within another cryptoendolithic fungus, *F. endolithicus*, isolated from Antarctica under γ-radiation stress (Pacelli et al. 2018b). These results may be attributed to the interaction of radiation with melanin reflected by the XTT assay and the increase in cell metabolic rates in response to radiation insult, perhaps resulting in damage repair (Pacelli et al. 2017a, 2017c, 2018a).

Notably, the highly damaging deuteron dosage caused a decrease in ATP levels in both melanised cells and non-melanised cells, with a sharper gradient observed in the melanised cells of *C. antarcticus* (Pacelli, Bryan, et al. 2017). A consistent drop in the ATP pattern was observed when *C. neoforman*s forming induced DOPA-melanin was exposed to a series of emission spectra from visible light to nonionising UV radiation and ionising gamma radiation (Bryan et al. 2011). Meanwhile, a slight decline was detected in non-melanised cells of *C. antarcticus* (Pacelli, Bryan, et al. 2017), while there was maintenance or even an increase in non-melanized *C. neoforman*s cells (Bryan et al. 2011; Pacelli et al. 2017a). An interesting possibility could be that melanins have functions analogous to other energy harvesting pigments, such as chlorophylls, resulting in ATP consumption during the synthesis of simple sugars by melanised fungi (Bryan et al. 2011). In fact, it is common to enhance the growth of melanised fungal cells after exposure to ionising radiation, such as black yeasts *Wangiella dermatitidis* and pathogenic *C. neoformans* (Dadachova et al. 2007). Apart from radiant energy transduction by melanin, it was also proposed that upregulation of many key genes caused by radiation stimulation and an inducible microhomology-mediated recombination pathway could be a potential mechanism of adaptative evolution in eukaryotes (Dadachova and Casadevall 2008).

Extremely slow-growing RIF has resulted in difficulties in performing deep investigation of their molecular mechanisms. However, the efficient genetic manipulation of the RIF strain *Knufia petricola* A95, a model species with the most typical characteristics of RIF that is widespread in most rock niches (Nai 2014), has been successfully established with a protoplast-based DNA transfer system (Noack-Schönmann et al. 2014). Traditional gene knockout, editing and replacement via plasmid-based or ribonucleoprotein (RNP)-based CRISPR/Cas9 and silencing by RNA interference (RNAi) have been realized (Voigt et al. 2020). These results certainly provide a new approach for adaptation mechanism studies of RIF to stresses.

## 5. Ecological significance and biotechnological exploration

### 5.1. Astrobiology and extraterrestrial life applications

Bare rocks represent a reasonably complicated ecological niche that is the closest to Mars-like conditions on Earth, especially in the McMurdo Dry Valleys, which is characterised by extremely hard desiccation, high UV exposure, extremely low temperatures and wide thermal fluctuations in the Antarctic (Onofri et al. 2004, 2007). It is thus of great significance to select rock-inhabiting fungi as eukaryotic models in astrobiology to investigate the possibility of extinct or extant life on extraterrestrial planets such as Mars. Melanin, with the detection of its clear and strong Raman signal (Culka et al. 2017), has been designated as a potential feasible biosignature by the BIOlogy and Mars EXperiment (BIOMEX), which aims to detect signatures of extinct or extant life using sensitive and nondestructive approaches (Selbmann et al. 2015; Coleine et al. 2021).

In the framework of the Lichens and Fungi Experiments (LIFE) programme conducted by the European Space Agency (Onofri et al. 2008), the Antarctic cryptoendolithic black fungal strain *C. antarcticu* CCFEE 515 exhibited great resistance with 12.5% viability of culturable cells and more than 60% of the cells remaining intact after long-term (1.5 years) exposure to the Simulated Martian Condition imitation experiment on the International Space Station (ISS) (Onofri et al. 2012, 2015), with only slight ultrastructural and molecular damage (Pacelli et al. 2017a, 2017c; Onofri et al. 2018). Concerning exposure to space-relevant irradiation, UV radiation, and even sparsely and densely ionising gamma, deuteron and X-ray radiation, *C. antarcticus* CCFEE 515 also exhibited striking endurance (Selbmann et al. 2018; Pacelli et al. 2019).

### 5.2. Biodeterioration and biogeochemistry

Fungi have been considered significant “invaders” (Vázquez-Nion et al. 2016; Pinheiro et al. 2019) that cause aesthetic, chemical, physical and mechanical deterioration of stones and especially rocky cultural relics such as sculptures, monuments and reliefs. Rock-inhabiting fungi were proven to be a more difficult enemy due to their excellent stress resistance and ability to perennially infect exposed bare rocks (Gorbushina 2007) and tenaciously recolonise stone walls after treatment with biocides or radiation (Sterflinger and Piñar 2013).

It was determined that biodeterioration mechanisms for RIF should involve mechanical destruction through hyphal penetration rather than the general acid dissolution mechanism used by many other types of fungi (Gorbushina et al. 1993). RIF tend to search for cavities or cause micropits on rock surfaces, forming a habitat or shelter to contain fungal colonies (Gadd 2017). These microcolonial fungi also developed an endolithic or chasmoendolithic ability to grow in cracks and pores (Caneva et al. 2014) by observation of junctions between crystals or thigmotropic penetration in weak areas (Scheerer et al. 2009). When subjected to extremely hostile conditions, they disappear from the surface and struggle to dig into sedimentary soft rock substrates such as carbonate at depths ranging from a few hundred microns to several millimetres for new substrates (Favero-Longo et al. 2011). It has been confirmed that fungal growth exerts a strong mechanical pressure, up to 12.39 bar, equal to 4.5 times the pressure that a person would require to crush a glass bottle (Dornieden et al. 2000; Bogomolova et al. 2003). Additionally, melanin confers extramechanical strength to the hyphae and enhances mechanical penetration (Onofri et al. 2014). Indeed, the chemically corrosive capacity of RIF cannot be ignored, including the secretion of siderophore-like compounds causing increased dissolution of limestone by the model iron chelator desferrioxamine (Favero-Longo et al. 2011).

RIF are also able to form biofilms in cooperation with other organisms, such as bacteria or algae (Seiffert et al. 2014). Subaerial biofilms (SABs) are composed mainly of phototrophic algae, cyanobacteria, heterotrophic bacteria and black rock fungi (Noack-Schönmann et al. 2014b). It has been frequently reported that SABs growing on solar panels may lead to a severe blockage of up to 70% of light transmission (Noack-Schönmann et al. 2014b; Shirakawa et al. 2015). During biofilm development processes, RIF are considered secondary residents, in contrast to the fast colonisation of rapid-growing pioneer colonisers, mainly Chlorophyta and Cyanobacteria, at the cost of a high organismal loss rate, with slower growth but more sustainable colonisation ability to cause low-speed but persistent material losses (Bogomolova et al. 2003; Vázquez-Nion et al. 2016).

### 5.3. Biotechnological exploration

Research on RIF has been carried out for broad biotechnological applications in the manufacturing, electronics engineering, astronautics, cosmetic, biomaterials, pharmaceutical and environmental bioremediation industries. Melanin is one of the most applicable materials derived from RIF.

To achieve good ultraviolet absorption properties, fungal melanin can be added as a protective component in the manufacture of blinkers, windows, packaging material, umbrellas, canopies (Pombeiro-Sponchiado et al. 2017), and even some skin photoprotection formulations such as face and hand creams, lotions, antiaging ointments, or foundation makeup (Liberti et al. 2020). Additionally, the capacity of melanin to attenuate ionising radiation, such as beta-radiation in outer space, holds great significance in radiation shield design for human space flight in general and habitat structures on the Moon and Mars (Lakk et al. 2018; Vasileiou and Summerer 2020). Considered an organic semiconductor that is cheaper and easier to process than inorganic semiconductors due to its similarity to amorphous semiconductor solids in terms of electrical conductivity properties, melanin could be a promising material for sensors and photovoltaic devices (Vahidzadeh et al. 2018).

Melanin was also proven sufficiently biocompatible to be applied as a nanocarrier drug release device during the treatment of intestinal and colon diseases or radiation therapy to tumours (Araújo et al. 2014). It is pleasantly surprising that some black fungi showed strong biological activities against the pathogenic bacteria *Staphylococcus aureus* and *Escherichia coli*, pathogenic fungi *Candida albicans* and *C. glabrata*, and even breast tumour cells (Gonçalves et al. 2014), indicating potential sources of bioactive compounds for drug discovery.

Given the ability of melanin to bind to metals and degrade exotic carbohydrates, black rock fungi could be good candidates for environmental bioremediation in contaminated sites with heavy metals and radionuclides, for example, to absorb or remove harmful volatile chemicals in decorated rooms (Prenafeta-Boldú et al. 2019) or to clean up industrial effluent with radioactive contamination (Singleton and Tobin 1996).

## 6. Conclusion and future remarks

Despite their sparse colonisation on terrestrial lands and slow growth on nutrient culture dishes, melanised microcolnial fungi inhabiting bare rocks, the “dark matter” in the world of eukaryotic life, have been unveiled to show formidable vitality, amazing biodiversity, momentous evolutionary status, extraordinary adaptation mechanisms, and promising biotechnological exploration.

Approaches based on culture-dependent and multilocus sequences have revealed huge biological diversity and the great potential of novel RIF taxa and have contributed to understanding the richness and distribution of RIF on rocks, especially cryptoendolithic communities in Antarctica (Coleine et al. 2021), in recent decades. High-throughput amplicon sequencing, as a new culture-independent method, has significantly enhanced the fungal communities in rock. However, there are some weaknesses in RIF community studies, such as exogenous DNA contamination of low-biomass assemblages (Cuscó et al. 2018) and limited identification by short amplicon regions such as ITS1 or ITS2 (Luo et al. 2020; Nagano et al. 2020; Zhang et al. 2020). The application of full-length amplicon sequencing by single-molecule real-time (SMRT) sequencing methods for species-level analysis (Zhang et al. 2020) might boost progress on the species diversity of RIF. Moreover, shotgun metagenomics and a single-cell genomics could also be a powerful means to detect novel taxa (Wu et al. 2019).

Although the term “rock-inhabing fungi” has been well accepted by mycologists and “lithophilic fungi” and “lithotolerant fungi” are also proposed in this paper, a thorough description and comprehensive understanding of fungi on rocks remain to be provided in future studies. Primarily, what is the boundary of rock-inhabiting fungi? In the “lithophilic and lithotolerant fungi” classification system of RIF, lithophilic fungi with typical RIF traits can be regarded as “kernels”, while lithotolerant fungi can be regarded as a “border” with polyextremotolerance of rock-like niches, since they play an ambiguous role between rock inhabitants and contamitants. Furthermore, is there a succinct parameter distinguishing lithophilic fungi and lithotolerant fungi? In general, the tiny colonies on rocks exhibit two extension forms on agar culture: extremely slow-growing microcolonies and moderate fast-growing ones (Table 1; Figure 1). The ratio of the growth on rock to that on agar culture should be more accurately evaluated and calculated, and a clear parameter might be deduced, which should be considered a meaningful attempt to delimit their true characteristics.

Now that this ecological fungal group is mainly affiliated with two classes, *Dothideomycetes* and *Eurotiomycetes*, and is distributed in different families and genera, similar adaptation to rock niches by different lineages of RIF might underlie their convergent evolution. Cellular adaptation to the rock niche may occur with different patterns; for example, phenotypic variation, such as the accumulation of chemical compounds and control of membrane fluidity, is easy and nonheritable (Naranjo‐Ortiz and Gabaldón 2019), and the evolution of genomes and transcription regulation styles is more profound. In the case of higher eukaryotes, a general stress response was observed when compared to different environmental stresses, such as light, heat or salt, underlying commonalities among diverse biotic stimuli (Cole and Tringe 2021). Unlike the HSP response common in fungal cells in response to stressful stimuli, the extraordinary proteomic dynamics of RIF indicate a potential specific adaptation mechanism following exposure to multiple and fluctuating stresses.

Rock-inhabiting fungi represent a broader research field and will provide a deeper understanding of eukaryotic organisms. Taxonomic studies tremendously enrich the biological diversity of fungi, ecology and evolution studies contribute to the inferrence of species origins, and exploration of adaptation mechanisms triggers the elucidation of antistress biology and cosmobiology. RIF is also a good example of resource utilisation from natural environments with broad applications in medicine, industry, and agriculture.

## 7. Species Checklist

**Typical rock-inhabiting fungi**

***Ascomycota***

***incertae sedis***

***Knufia*** L.J. Hutchison & Unter., Mycologia 87: 903 (1996)

***Knufia calcicola*** L. Su, W. Sun & M.C. Xiang, Journal of Fungi 6 (4, no. 187): 18 (2020)

Obligate synonyms: ***Knufia calcarecola*** L. Su, W. Sun & M.C. Xiang (2020) Orthographic variant

***Knufia karalitana*** Isola & Onofri, Fungal Systematics and Evolution 3: 128 (2019)

Taxon synonyms: ***Knufia karalitana*** Isola & Onofri, Fungal Diversity 76: 88 (2015) invalid Art. 40.7 (Melbourne)

***Knufia marmoricola*** Onofri & Zucconi, Fungal Systematics and Evolution 3: 128 (2019)

Taxon synonyms: ***Knufia marmoricola*** Onofri & Zucconi, Fungal Diversity 76: 88 (2015) invalid Art. 40.7 (Melbourne)

***Knufia mediterranea*** Selbmann & Zucconi, Fungal Systematics and Evolution 3: 128 (2019)

***Knufia separata*** L. Su, W. Sun & M.C. Xiang, Journal of Fungi 6 (4, no. 187): 19 (2020)

***Knufia vaticanii*** Zucconi & Onofri, Fungal Diversity 76: 88 (2015) invalid Art. 40.7 (Melbourne)

***Knufia perforans*** (Sterfl.) Tsuneda, Hambl. & Currah, Botany 89: 887 (2011)

Obligate synonyms: ***Knufia perforans*** (Sterfl.) Tsuneda, Hambl. & Currah, Botany 89 (8): 534 (2011) Invalid Art. 41.5 (Melbourne)

Basionym: ***Coniosporium perforans*** Sterfl., Antonie van Leeuwenhoek 72 (4): 352 (1997)

***Knufia petricola*** (Wollenz. & de Hoog) Gorbushina & Gueidan, Fungal Genetics & Biology 56: 58 (2013)

Basionym: ***Sarcinomyces petricola*** Wollenz. & de Hoog, Antonie van Leeuwenhoek 71 (3): 283 (1997)

***Knufia chersonesos*** (Bogomolva & Minter) Tsuneda, Hambl. & Currah, Botany 89: 887 (2011)

Basionym: ***Phaeococcomyces chersonesos*** Bogom. & Minter, Mycotaxon 86: 203 (2003)

Obligate synonyms: ***Knufia chersonesos*** (Bogomolva & Minter) Tsuneda, Hambl. & Currah, Botany 89 (8): 535 (2011) Invalid

***Dothideomycetes***

***incertae sedis***

***Cryomyces*** Selbmann, de Hoog, Mazzaglia, Friedmann & Onofri, Studies in Mycology 51: 19 (2005)

***Cryomyces antarcticus*** Selbmann, de Hoog, Mazzaglia, Friedmann & Onofri, Studies in Mycology 51: 19 (2005)

***Cryomyces funiculosus*** Selbmann & de Hoog, Fungal Diversity 86: 123 (2017)

Taxon synonyms: ***Cryomyces funiculosus*** Selbmann & de Hoog, Fungal Diversity 65 (1): 175 (2013) Invalid Art. 40.6

***Cryomyces minteri*** Selbmann, de Hoog, Mazzaglia, Friedmann & Onofri, Studies in Mycology 51: 21 (2005)

***Cryomyces montanus*** Isola & Zucconi, Fungal Diversity 86: 123 (2017)

Taxon synonyms: ***Cryomyces montanus*** Isola & Zucconi, Fungal Diversity 65 (1): 177 (2013) Invalid Art. 40.6

***Phaeosclera*** Sigler, Tsuneda & J.W. Carmich., Mycotaxon 12 (2): 461 (1981)

***Rupestriomyces*** L. Su, L.Y. Guo & X.Z. Liu, Mycologia 107 (4): 839 (2015)

***Rupestriomyces ampulliformis*** L. Su, L.Y. Guo & X.Z. Liu, Mycologia 107 (4): 841 (2015)

***Rupestriomyces sinensis*** L. Su, L.Y. Guo & X.Z. Liu, Mycologia 107 (4): 840 (2015)

***Rupestriomyces torulosus*** L. Su, L.Y. Guo & X.Z. Liu, Mycologia 107 (4): 840 (2015)

***Saxomyces*** Selbmann & Isola, Fungal Diversity 86: 422 (2017)

Taxon synonyms: ***Saxomyces*** Selbmann & Isola, Fungal Diversity 65 (1): 174 (2013) Invalid Art. 40.1

***Saxomyces alpinus*** Zucconi & Selbmann, Fungal Diversity 86: 422 (2017)

Taxon synonyms: ***Saxomyces alpinus*** Zucconi & Selbmann, Fungal Diversity 65 (1): 174 (2013) Invalid Art. 40.6

***Saxomyces penninicus*** Zucconi & Onofri, Fungal Diversity 86: 422 (2017)

Taxon synonyms: ***Saxomyces penninicus*** Zucconi & Onofri, Fungal Diversity 65 (1): 175 (2013) Invalid Art. 40.6

***Spissiomyces*** Lei Su, Li Y. Guo & Xing Z. Liu, Mycologia 107 (4): 837 (2015)

***Spissiomyces aggregatus*** Lei Su, Li Y. Guo & Xing Z. Liu, Mycologia 107 (4): 838 (2015)

***Spissiomyces ramosus*** Lei Su, Li Y. Guo & Xing Z. Liu, Mycologia 107 (4): 838 (2015)

***Coniosporiales***

***Coniosporiaceae***

***Coniosporium*** Link, Magazin der Gesellschaft Naturforschenden Freunde Berlin 3 (1): 8 (1809)

Taxon synonyms: ***Conisporium*** Link, Magazin der Gesellschaft Naturforschenden Freunde Berlin 3 (1): 8 (1809) Orthographic variant

***Coniosporium apollinis*** Sterfl., Antonie van Leeuwenhoek 72 (4): 358 (1997)

***Coniosporium sümbülii*** Sert & Sterflinger, Mycological Progress 9 (3): 356 (2010)

***Coniosporium uncinatum*** De Leo, Urzì & de Hoog, Studies in Mycology 43: 75 (1999)

***Dothideomycetidae***

***Capnodiales***

***incertae sedis***

***Arthrocatena*** Egidi & Selbmann, Fungal Systematics and Evolution 3: 126 (2019)

Taxon synonyms: ***Arthrocatena*** Egidi & Selbmann, Fungal Diversity 65: 159 (2014) Invalid Art. 40.7 (Shenzhen)

***Arthrocatena tenebrosa*** Egidi & Selbmann, Fungal Systematics and Evolution 3: 126 (2019)

Taxon synonyms: ***Arthrocatena tenebrio*** Egidi & Selbmann, Fungal Diversity 65: 159 (2014) Invalid Art. 40.7 (Shenzhen)

***Capnobotryella*** Sugiy., Pleomorphic Fungi: The Diversity and its Taxonomic Implications (Tokyo): 148 (1987)

***Capnobotryella antalyensis*** Sert & Sterflinger, Mycological Research 111 (10): 1237 (2007)

***Capnobotryella renispora*** Sugiy., Two metacapnodiaceous sooty moulds from Japan: their identity and behaviour in pure culture: 148 (1987)

***Capnobotryella erdogani***

***Capnobotryella isiloglui***

***Capnobotryella kiziroglui***

***Catenulomyces*** Egidi & de Hoog, Fungal Systematics and Evolution 3: 126 (2019)

Taxon synonyms: ***Catenulomyces*** Egidi & de Hoog, Fungal Diversity 65: 154 (2014) Invalid Art. 40.7 (Shenzhen)

***Catenulomyces convolutus*** Egidi & de Hoog, Fungal Systematics and Evolution 3: 126 (2019)

Taxon synonyms: ***Catenulomyces convolutus*** Egidi & de Hoog, Fungal Diversity 65: 154 (2014) Invalid Art. 40.7 (Shenzhen)

***Constantinomyces*** Egidi & Onofri, Fungal Systematics and Evolution 3: 126 (2019)

Taxon synonyms: ***Constantinomyces*** Egidi & Onofri, Fungal Diversity 65: 155 (2014) Invalid Art. 40.7 (Shenzhen)

***Constantinomyces macerans*** de Hoog & Onofri, Fungal Systematics and Evolution 3: 126 (2019)

Taxon synonyms: ***Constantinomyces macerans*** de Hoog & Onofri, Fungal Diversity 65: 157 (2014) Invalid Art. 40.7 (Shenzhen)

***Constantinomyces minimus*** de Hoog & Isola, Fungal Systematics and Evolution 3: 126 (2019)

Taxon synonyms: ***Constantinomyces minimus*** de Hoog & Isola, Fungal Diversity 65: 157 (2014) Invalid Art. 40.7 (Shenzhen)

***Constantinomyces nebulosus*** Isola & Zucconi, Fungal Systematics and Evolution 3: 126 (2019)

Taxon synonyms: ***Constantinomyces nebulosus*** Isola & Zucconi, Fungal Diversity 65: 157 (2014) Invalid Art. 40.7 (Shenzhen)

***Constantinomyces oldenburgensis*** Gorbushina, P.M. Martin-Sanchez, Ruibal & Selbmann, Life 8 (3/30): 8 (2018) Invalid Art. 40.7 (Shenzhen)

***Constantinomyces patonensis*** Ruibal & Selbmann, Life 8 (3/30): 11 (2018) Invalid Art. 40.7 (Shenzhen)

***Constantinomyces virgultus*** Egidi & Onofri, Fungal Systematics and Evolution 3: 127 (2019)

Taxon synonyms: ***Constantinomyces virgultus*** Egidi & Onofri, Fungal Diversity 65: 155 (2014) Invalid Art. 40.7 (Shenzhen)

***Elasticomyces*** Zucconi & Selbmann, Studies in Mycology 61: 11 (2008)

***Elasticomyces elasticus*** Zucconi & Selbmann, Studies in Mycology 61: 11 (2008)

***Friedmanniomyces*** Onofri, Nova Hedwigia 68: 176 (1999)

***Friedmanniomyces endolithicus*** Onofri, Nova Hedwigia 68: 177 (1999)

***Friedmanniomyces simplex*** Selbmann, de Hoog, Mazzaglia, Friedmann & Onofri, Studies in Mycology 51: 16 (2005)

***Hyphoconis*** Egidi & Quaedvl., Fungal Systematics and Evolution 3: 127 (2019)

Taxon synonyms: ***Hyphoconis*** Egidi & Quaedvl., Fungal Diversity 65: 153 (2014) Invalid Art. 40.7 (Shenzhen)

***Hyphoconis sterilis*** Egidi & Quaedvl., Fungal Systematics and Evolution 3: 127 (2019)

Taxon synonyms: ***Hyphoconis sterilis*** Egidi & Quaedvl., Fungal Diversity 65: 153 (2014) Invalid Art. 40.7 (Shenzhen)

***Incertomyces*** Egidi & Zucconi, Fungal Systematics and Evolution 3: 127 (2019)

Taxon synonyms: ***Incertomyces*** Egidi & Zucconi, Fungal Diversity 65: 157 (2014) Invalid Art. 40.7 (Shenzhen)

***Incertomyces perditus*** Egidi & Zucconi, Fungal Systematics and Evolution 3: 127 (2019)

Taxon synonyms: ***Incertomyces perditus*** Egidi & Zucconi, Fungal Diversity 65: 157 (2014) Invalid Art. 40.7 (Shenzhen)

***Incertomyces vagans*** Egidi & Selbmann, Fungal Diversity 65: 157 (2014)

***Lapidomyces*** de Hoog & Stielow, Fungal Systematics and Evolution 3: 128 (2019)

Taxon synonyms: ***Lapidomyces*** de Hoog & Stielow, Fungal Diversity 65: 159 (2014) Invalid Art. 40.7 (Shenzhen)

***Lapidomyces hispanicus*** de Hoog & Stielow, Fungal Systematics and Evolution 3: 128 (2019)

Taxon synonyms: ***Lapidomyces hispanicus*** de Hoog & Stielow, Fungal Diversity 65: 159 (2014) Invalid Art. 40.7 (Shenzhen)

***Meristemomyces*** Isola & Onofri, Fungal Systematics and Evolution 3: 128 (2019)

Taxon synonyms: ***Meristemomyces*** Isola & Onofri, Fungal Diversity 65: 158 (2014) Invalid Art. 40.1, see Arts 40.3 and Arts 6.3, 12.1 (Melbourne)

***Meristemomyces frigidus*** Isola & Onofri, Fungal Systematics and Evolution 3: 129 (2019)

Taxon synonyms: ***Meristemomyces frigidus*** Isola & Onofri, Fungal Systematics and Evolution 3: 129 (2019) Invalid Art. 40.7 (Shenzhen)

***Monticola*** Selbmann & Egidi, Fungal Systematics and Evolution 3: 128 (2019)

Taxon synonyms: ***Monticola*** Selbmann & Egidi, Fungal Diversity 65: 155 (2014) Invalid Art. 40.1, see Arts 40.3 and Arts 6.3, 12.1 (Melbourne)

***Monticola elongata*** Selbmann & Egidi, Fungal Systematics and Evolution 3: 128 (2019)

Taxon synonyms: ***Monticola elongata*** Selbmann & Egidi, Fungal Diversity 65: 155 (2014) Invalid Art. 40.7 (Melbourne)

***Oleoguttula*** Selbmann & de Hoog, Fungal Systematics and Evolution 3: 129 (2019)

Taxon synonyms: ***Oleoguttula*** Selbmann & de Hoog, Fungal Diversity 65: 152 (2014) Invalid Art. 40.1, see Arts 40.3 and Arts 6.3, 12.1 (Melbourne)

***Oleoguttula mirabilis*** Selbmann & de Hoog, Fungal Systematics and Evolution 3: 129 (2019)

Taxon synonyms: ***Oleoguttula mirabilis*** Selbmann & de Hoog, Fungal Systematics and Evolution 3: 129 (2019) Invalid Art. 40.7 (Shenzhen)

***Penidiella*** Crous & U. Braun, Studies in Mycology 58: 17 (2007)

***Penidiella ellipsoidea*** Crous, Persoonia 26: 78 (2011)

***Perusta*** Egidi & Stielow, Fungal Systematics and Evolution 3: 130 (2019)

Taxon synonyms: ***Perusta*** Egidi & Stielow, Fungal Diversity 65: 155 (2014) Invalid Art. 40.1 (Shenzhen)

***Perusta inaequalis*** Egidi & Stielow, Fungal Systematics and Evolution 3: 130 (2019)

Taxon synonyms: ***Perusta inaequalis*** Egidi & Stielow, Fungal Diversity 65: 155 (2014) Invalid Art. 40.7 (Shenzhen)

***Petrophila*** de Hoog & Quaedvl., Fungal Systematics and Evolution 3: 130 (2019)

Taxon synonyms: ***Petrophila*** de Hoog & Quaedvl., Fungal Diversity 65: 152 (2014) Invalid Art. 40.1, see Arts 40.3 and Arts 6.3, 12.1 (Shenzhen)

***Petrophila incerta*** de Hoog & Quaedvl., Fungal Systematics and Evolution 3: 130 (2019)

Taxon synonyms: ***Petrophila incerta*** de Hoog & Quaedvl., Fungal Diversity 65: 152 (2014) Invalid Art. 40.7 (Shenzhen)

***Pseudotaeniolina*** J.L. Crane & Schokn., Mycologia 78 (1): 88 (1986)

***Pseudotaeniolina globosa*** De Leo, Urzì & de Hoog, Antonie van Leeuwenhoek 83 (4): 356 (2003)

***Ramimonilia*** Stielow & Quaedvl., Fungal Systematics and Evolution 3: 130 (2019)

Taxon synonyms: ***Ramimonilia*** Stielow & Quaedvl., Fungal Diversity 65: 155 (2014) Invalid Art. 40.1, see Arts 40.3 and Arts 6.3, 12.1 (Shenzhen)

***Ramimonilia apicalis*** Stielow & Quaedvl., Fungal Systematics and Evolution 3: 131 (2019)

Taxon synonyms: ***Ramimonilia apicalis*** Stielow & Quaedvl., Fungal Diversity 65: 155 (2014) Invalid Art. 40.7 (Shenzhen)

***Recurvomyces*** Selbmann & de Hoog, Studies in Mycology 61: 10 (2008)

***Recurvomyces mirabilis*** Selbmann & de Hoog, Studies in Mycology 61: 11 (2008)

***Saxophila*** Selbmann & de Hoog, Fungal Systematics and Evolution 3: 131 (2019)

Taxon synonyms: ***Saxophila*** Selbmann & de Hoog, Fungal Diversity 76: 90 (2015) Invalid Art. 40.1 (Shenzhen)

***Saxophila tyrrhenica*** Selbmann & de Hoog, Fungal Systematics and Evolution 3: 131 (2019)

Taxon synonyms: ***Saxophila tyrrhenica*** Selbmann & de Hoog, Fungal Diversity 76: 90 (2015) Invalid Art. 40.7 (Shenzhen)

***Vermiconidia*** Egidi & Onofri, Fungal Systematics and Evolution 3: 131 (2019)

Taxon synonyms: ***Vermiconia*** Egidi & Onofri, Fungal Diversity 65: 150 (2014) Invalid Art. 40.1, see Arts 40.3 and Arts 6.3, 12.1 (Shenzhen)

***Vermiconidia antarctica*** Egidi & Selbmann, Fungal Systematics and Evolution 3: 132 (2019)

Taxon synonyms: ***Vermiconia antarctica*** Egidi & Selbmann, Fungal Diversity 65: 152 (2014) Invalid Art. 40.7 (Shenzhen)

***Vermiconidia calcicola*** de Hoog & Onofri, Fungal Systematics and Evolution 3: 132 (2019)

Taxon synonyms: ***Vermiconia calcicola*** de Hoog & Onofri, Fungal Diversity 76: 90 (2015) Invalid Art. 40.7 (Shenzhen)

***Vermiconidia flagrans*** Selbmann & Isola, Fungal Systematics and Evolution 3: 132 (2019)

Taxon synonyms: ***Vermiconia flagrans*** Selbmann & Isola, Fungal Diversity 65: 152 (2014) Invalid Art. 40.7 (Shenzhen)

***Vermiconidia foris*** Egidi & Onofri, Fungal Systematics and Evolution 3: 132 (2019)

Taxon synonyms: ***Vermiconia foris*** Egidi & Onofri, Fungal Diversity 65: 150 (2014) Invalid Art. 40.7 (Shenzhen)

***Aeminiaceae***

***Aeminiaceae*** J. Trovão, I. Tiago & A. Portugal, MycoKeys 45: 62 (2019)

***Aeminium*** J. Trovão, I. Tiago & A. Portugal, MycoKeys 45: 64 (2019)

***Aeminium ludgeri*** J. Trovão, I. Tiago & A. Portugal, MycoKeys 45: 64 (2019)

***CapnodiaceaeLeptoxyphium*** Speg., Physis Revista de la Sociedad Argentina de Ciencias Naturales 4 (17): 294 (1918)

Taxon synonyms: ***Astragoxyphium*** Bat., Nascim. & Cif., Quaderno del Laboratorio Crittogamico del Istituto Botanico dell’Università di Pavia 31: 45 (1963)

Taxon synonyms: ***Megaloxyphium*** Cif., Bat. & Nascim., Publicações do Instituto de Micologia da Universidade do Recife 47: 3 (1956)

***ParadevriesiaceaeParadevriesia*** Crous, Fungal Systematics and Evolution 3: 98 (2019)

***Paradevriesia compacta*** Crous, Fungal Systematics and Evolution 3: 129 (2019)

Taxon synonyms: ***Devriesia compacta*** de Hoog & Quaedvl., Fungal Diversity 65: 148 (2014) Invalid Art. 40.7 (Shenzhen)

***TeratosphaeriaceaeAcrodontium*** de Hoog, Studies in Mycology 1: 23 (1972)

***Acrodontium crateriforme*** (J.F.H. Beyma) de Hoog, Studies in Mycology 1: 26 (1972)

Basionym: ***Chloridium crateriforme*** J.F.H. Beyma, Zentralblatt für Bakteriologie und Parasitenkunde, Abteilung 2 89: 241 (1933)

Obligate synonyms: ***Tritirachium crateriforme*** (J.F.H. Beyma) Matsush., Icones Microfungorum a Matsushima lectorum: 160 (1975)

***Austroafricana*** Quaedvl. & Crous, Persoonia 33: 25 (2014)

***Austroafricana parva*** (R.F. Park & Keane) Quaedvl. & Crous, Persoonia 33: 25 (2014)

Basionym: ***Mycosphaerella parva*** R.F. Park & Keane, Transactions of the British Mycological Society 79 (1): 99 (1982)

Obligate synonyms: ***Teratosphaeria parva*** (R.F. Park & Keane) Crous & U. Braun, Studies in Mycology 58: 10 (2007)

Taxon synonyms: ***Mycosphaerella grandis*** Carnegie & Keane, Mycological Research 98: 414 (1994)

***Catenulostroma*** Crous & U. Braun, Studies in Mycology 58: 13 (2007)

***Catenulostroma protearum*** (Crous & M.E. Palm) Crous & U. Braun, Studies in Mycology 58: 17 (2007)

Basionym: ***Trimmatostroma protearum*** Crous & M.E. Palm, Mycological Research 103 (10): 1303 (1999)

***Hortaea*** Nishim. & Miyaji, Japanese Journal of Medical Mycology 26 (2): 145 (1984)

***Hortaea thailandica*** Crous & K.D. Hyde, Studies in Mycology 64: 39 (2009)

***Hortaea werneckii*** (Horta) Nishim. & Miyaji, Japanese Journal of Medical Mycology 26 (2): 145 (1984)

Basionym: ***Cladosporium werneckii*** Horta, Revista Med. Cirurgía Brasil 29: 274 (1921)

Obligate synonyms: ***Exophiala werneckii*** (Horta) Arx, The genera of fungi sporulating in pure culture: 180 (1970)

Obligate synonyms: ***Pullularia werneckii*** (Horta) G.A. de Vries, Contribution to the knowledge of the genus Cladosporium: 101 (1952)

Obligate synonyms: ***Phaeoannellomyces werneckii*** (Horta) McGinnis & Schell, Sabouraudia 23: 184 (1979)

Obligate synonyms: ***Dematium werneckii*** (Horta) C.W. Dodge, Medical mycology. Fungous diseases of men and other mammals: 676 (1935)

Taxon synonyms: ***Cladosporium rietmanni*** Sartory, Rev. Pat. Malad. Pays Chauds: 9–44 (1935)

Taxon synonyms: ***Pullularia fermentans var. leaoi*** E.S. Wynne & Gott, Journal of General Microbiology 14: 517 (1956)

Taxon synonyms: ***Cryptococcus metaniger*** Castell., Archives of Dermatology and Syphilology 16 (4): 402 (1927)

Taxon synonyms: ***Cladosporium metaniger*** (Castell.) Ferrari, Atti dell’Istituto Botanico della Università e Laboratorio Crittogamico di Pavia 3: 183 (1932)

Taxon synonyms: ***Pullularia fermentans var. castellanii*** E.S. Wynne & Gott, Journal of General Microbiology 14: 518 (1956)

Taxon synonyms: ***Circinotrichum metaniger*** (Castell.) M. Ota, C.R. Soc. Biol. Paris: 1187 (1936)

***Neocatenulostroma*** Quaedvl. & Crous, Persoonia 33: 26 (2014)

***Neocatenulostroma abietis*** (Butin & Pehl) Quaedvl. & Crous, Persoonia 33: 27 (2014)

Basionym: ***Trimmatostroma abietis*** Butin & Pehl, Antonie van Leeuwenhoek 69 (3): 204 (1996)

Obligate synonyms: ***Catenulostroma abietis*** (Butin & Pehl) Crous & U. Braun, Studies in Mycology 58: 15 (2007)

***Cladosporiales***

***Cladosporiaceae***

***Rachicladosporium*** Crous, U. Braun & C.F. Hill, Studies in Mycology 58: 38 (2007)

***Rachicladosporium alpinum*** Egidi & Zucconi, Fungal Systematics and Evolution 3: 130 (2019)

Taxon synonyms: ***Rachicladosporium alpinum*** Egidi & Zucconi, Fungal Diversity 65: 159 (2014) Invalid Art. 40.7 (Shenzhen)

***Rachicladosporium antarcticum*** Onofri & Egidi, Fungal Diversity 65: 162 (2014)

***Rachicladosporium inconspicuum*** de Hoog & Stielow, Fungal Systematics and Evolution 3: 130 (2019)

Taxon synonyms: ***Rachicladosporium inconspicuum*** de Hoog & Stielow, Fungal Diversity 65: 162 (2014) Invalid Art. 40.7 (Shenzhen)

***Rachicladosporium mcmurdoi*** Selbmann & Onofri, Fungal Systematics and Evolution 3: 130 (2019)

Taxon synonyms: ***Rachicladosporium mcmurdoi*** Selbmann & Onofri, Fungal Diversity 65: 159 (2014) Invalid Art. 40.7 (Shenzhen)

***Rachicladosporium monterosanum*** Isola & Zucconi, Fungal Systematics and Evolution 3: 130 (2019)

Taxon synonyms: ***Rachicladosporium monterosium*** Isola & Zucconi, Fungal Diversity 65: 161 (2014) Invalid Art. 40.7 (Shenzhen)

***Rachicladosporium paucitum*** Isola & Egidi, Fungal Systematics and Evolution 3: 130 (2019)

Taxon synonyms: ***Rachicladosporium paucitum*** Isola & Egidi, Fungal Diversity 65: 162 (2014) Invalid Art. 40.7 (Shenzhen)

***Verrucocladosporium*** K. Schub., Aptroot & Crous, Studies in Mycology 58: 41 (2007)

***Verrucocladosporium dirinae*** K. Schub., Aptroot & Crous, Studies in Mycology 58: 41 (2007)

***Dothideales***

***Dothioraceae***

***Aureobasidium*** Viala & G. Boyer, Revue Génerale de Botanique 3: 371 (1891)

Obligate synonyms: ***Aureobasis*** Clem. & Shear, The genera of Fungi: 343, 381 (1931)

Obligate synonyms: ***Chrysobasidium*** Clem., The genera of Fungi: 107 (1909)

Taxon synonyms: ***Pullularia*** Berkhout, De schimmelgeslachten Monilia, Oidium, Oospora en Torula: 55, 64 (1923)

Taxon synonyms: ***Dematoidium*** Stautz, Phytopathologische Zeitschrift 3: 204 (1931)

Taxon synonyms: ***Pachybasidiella*** Bubák & Syd., Annales Mycologici 13 (1): 9 (1915)

Taxon synonyms: ***Protocoronospora*** G.F. Atk. & Edgerton, Journal of Mycology 13 (5): 186 (1907)

Taxon synonyms: ***Protocoronis*** Clem. & Shear, The genera of Fungi: 197, 344 (1931)

Taxon synonyms: ***Dematoideum*** Stautz (1931)

***Aureobasidium pullulans*** (de Bary) G. Arnaud, Annales de l’École Nationale d’Agriculture de Montpellier 16 (1–4): 39 (1918)

Basionym: ***Dematium pullulans*** de Bary, Vergleichende Morphologie und Biologie der Pilze Mycetozoen und Bacterien: 182 (1884) [MB#219317]

Obligate synonyms: ***Anthostomella pullulans*** (de Bary) F.T. Benn., Annals of Applied Biology 15: 381 (1928)

Obligate synonyms: ***Pullularia pullulans*** (de Bary) Berkhout, De schimmelgeslachten Monilia, Oidium, Oospora en Torula: 55 (1923)

Obligate synonyms: ***Hormonema pullulans*** (de Bary) Lagerb. & Melin, Nytt Magazin for Naturvidenskapene 71: 256 (1932)

Obligate synonyms: ***Cladosporium pullulans*** (de Bary) Sacc. & Trotter, Sylloge Fungorum 22: 1250 (1913)

Taxon synonyms: ***Aureobasidium vitis*** Viala & G. Boyer, Revue Génerale de Botanique 3: 371 (1891)

Taxon synonyms: ***Exobasidium vitis*** (Viala & G. Boyer) Prill. & Delacr. (1894) Taxon synonyms: Taxon synonyms: ***Chrysobasidium vitis*** (Viala & G. Boyer) Clem., The genera of Fungi: 107 (1909)

Taxon synonyms: ***Aureobasis vitis*** (Viala & G. Boyer) Clem. & Shear, The genera of Fungi: 343, 381 (1931)

Taxon synonyms: ***Dematoidium nigrescens*** Stautz, Phytopathologische Zeitschrift 3: 204 (1931)

Taxon synonyms: ***Phymatotrichum baccarum*** Oudem., Verslag Verg. Afd. Natuurkunde KNAW: 392 (1900)

Taxon synonyms: ***Aureobasidium pullulans*** (De Bary) G. Arnaud ex Cif., Ribaldi & Corte, Atti dell’Istituto Botanico della Università e Laboratorio Crittogamico di Pavia 14: 85 (1957)

***Endoconidioma*** Tsuneda, Hambl. & Currah, Mycologia 96 (5): 1129 (2004)

Taxon synonyms: ***Coniozyma*** Crous, CBS Biodiversity Series 7: 97 (2008)

***Hormonema*** Lagerb. & Melin, Svenska Skogsvårdsföreningens Tidskrift 2 (2–4): 219 (1927)

***Hormonema carpetanum*** Bills, Peláez & Ruibal, Studies in Mycology 50 (1): 152 (2004)

***Pringsheimia*** Schulzer, Verhandlungen der Zoologisch-Botanischen Gesellschaft Wien 16: 57 (1866)

***Pringsheimia smilacis*** E. Müll., Sydowia 11: 458 (1957)

***Mycosphaerellales***

***Extremaceae***

***Extremus*** Quaedvl. & Crous, Fungal Systematics and Evolution 3: 127 (2019)

Taxon synonyms: ***Extremus*** Quaedvl. & Crous, Persoonia 33: 21 (2014) Invalid Art. 40.7 (Shenzhen)

***Extremus adstrictus*** Quaedvl. & Crous, Fungal Systematics and Evolution 3: 127 (2019)

Taxon synonyms: ***Extremus adstrictus*** Egidi & Onofri ex Quaedvl. & Crous, Persoonia 33: 22 (2014) Invalid Art. 40.7 (Shenzhen)

Taxon synonyms: ***Devriesia adstricta*** Egidi & Onofri, Fungal Diversity 65: 150 (2014)

***Extremus antarcticus*** Quaedvl. & Crous, Fungal Systematics and Evolution 3: 127 (2019)

Taxon synonyms: ***Extremus antarcticus*** Selbmann & de Hoog ex Quaedvl. & Crous, Persoonia 33: 22 (2014) Invalid Art. 40.7 (Shenzhen)

Taxon synonyms: ***Devriesia antarctica*** Selbmann & de Hoog, Fungal Diversity 65: 150 (2014)

***Mycosphaerellaceae***

***Pseudocercospora*** Speg., Anales del Museo Nacional de Historia Natural Buenos Aires ser. 3, 13: 438 (1911)

Taxon synonyms: ***Stigmina*** Sacc., Michelia 2 (6): 22 (1880)

Taxon synonyms: ***Ciferriella*** Petr., Annales Mycologici 28 (5–6): 409 (1930)

Taxon synonyms: ***Ancylospora*** Sawada, Report of the Department of Agriculture Government Research Institute of Formosa 87: 77 (1944)

Taxon synonyms: ***Cercocladospora*** G.P. Agarwal & S.M. Singh, Proc. natn. Acad. Sci. India, Sect. B, Biol. Sci.: 439 (1974)

Taxon synonyms: ***Cercosporiopsis*** Miura, Flora of Manchuria and East Mongolia. Part III. Cryptogams, fungi 3: 527–528 (1928)

Taxon synonyms: ***Helicomina*** L.S. Olive, Mycologia 40 (1): 16 (1948)

Taxon synonyms: ***Pseudocercospora*** sect. Helicomina (L.S. Olive) U. Braun, A monograph of Cercosporella, Ramularia and allied genera (phytopathogenic Hyphomycetes) 2: 398 (1998)

Taxon synonyms: ***Jaczewskiella*** Murashk., Mater. Mikol. Fitopat. Ross.: 5 (1926)

Taxon synonyms: ***Marcosia*** Syd. & P. Syd., Annales Mycologici 14 (1–2): 96 (1916)

Taxon synonyms: ***Pseudopuccinia*** Höhn., Mitt. bot. Inst. tech. Hochsch. Wien: 41 (1925)

Taxon synonyms: ***Semipseudocercospora*** J.M. Yen, Mycotaxon 17: 361 (1983)

Taxon synonyms: ***Pseudocercospora*** sect. Cercocladospora G.P. Agarwal & S.M. Singh ex U. Braun, A monograph of Cercosporella, Ramularia and allied genera (phytopathogenic Hyphomycetes) 2: 397 (1998)

Taxon synonyms: ***Neopseudocercospora*** Crous, Persoonia 31: 219 (2013)

***NeodevriesiaceaeNeodevriesia*** Quaedvl. & Crous, Persoonia 33: 24 (2014)

***Neodevriesia bulbillosa*** Egidi & Zucconi, Fungal Systematics and Evolution 3: 129 (2019)

Taxon synonyms: ***Neodevriesia bulbillosa*** E. Egidi & Zucconi ex Crous, Sydowia 67: 108 (2015) Invalid Art. 40.7 (Shenzhen)

Taxon synonyms: ***Devriesia bulbillosa*** Egidi & Zucconi, Fungal Diversity 65: 148 (2014) Invalid Art. 40.7 (Shenzhen)

***Neodevriesia capensis*** (Crous) Crous, Sydowia 67: 108 (2015)

Basionym: ***Teratosphaeria capensis*** Crous, Persoonia 27: 38 (2011)

***Neodevriesia lagerstroemiae*** (Crous & M.J. Wingf.) Crous, Sydowia 67: 108 (2015)

Basionym: ***Devriesia lagerstroemiae*** Crous & M.J. Wingf., Studies in Mycology 64: 38 (2009)

***Neodevriesia modesta*** Isola & Zucconi, Fungal Systematics and Evolution 3: 129 (2019)

Taxon synonyms: ***Neodevriesia modesta*** Isola & Zucconi ex Crous, Sydowia 67: 108 (2015) Invalid Art. 40.7 (Shenzhen)

Taxon synonyms: ***Devriesia modesta*** Isola & Zucconi, Fungal Diversity 65: 148 (2014) Invalid Art. 40.7 (Shenzhen)

***Neodevriesia sardiniae*** Isola & de Hoog, Fungal Systematics and Evolution 3: 129 (2019)

Taxon synonyms: ***Neodevriesia sardiniae*** D. Isola & de Hoog ex M.M. Wang & L. Cai, Mycologia 109 (6): 972 (2017) Invalid Art. 40.7 (Shenzhen)

Taxon synonyms: ***Devriesia sardiniae*** Isola & de Hoog, Fungal Diversity 76: 85 (2015) Invalid Art. 40.7 (Shenzhen)

***Neodevriesia simplex*** Selbmann & Zucconi, Fungal Systematics and Evolution 3: 129 (2019)

Taxon synonyms: ***Neodevriesia simplex*** Selbmann & Zucconi ex Crous, Sydowia 67: 108 (2015) Invalid Art. 40.7 (Shenzhen)

Taxon synonyms: ***Devriesia simplex*** Selbmann & Zucconi, Fungal Diversity 65: 148 (2014) Invalid Art. 40.7 (Shenzhen)

***Neophaeothecales***

***NeophaeothecaceaeNeophaeotheca*** Abdollahz. & Crous, Studies in Mycology 95: 392 (2020)

***Neophaeotheca triangularis*** (de Hoog & Beguin) Abdollahz. &Crous, Studies in Mycology 95: 392 (2020)

Basionym: ***Phaeotheca triangularis*** de Hoog & Beguin, Antonie van Leeuwenhoek 71 (3): 290 (1997)

***Phaeothecales***

***PhaeothecaceaePhaeotheca*** Sigler, Tsuneda & J.W. Carmich., Mycotaxon 12 (2): 450 (1981)

***Pleosporomycetidae***

***Venturiales***

***Sympoventuriaceae***

***Ochroconis*** de Hoog & Arx, Kavaka 1: 57 (1973)

***Ochroconis anomala*** A. Nováková & P.M. Martin-Sanchez, Fungal Biology 116 (5): 584 (2012)

Obligate synonyms: ***Scolecobasidium anomalum*** (A. Nováková & P.M. Martin-Sanchez) G.Y. Sun & Lu Hao, Fungal Biology 12: 491 (2013)

***Ochroconis lascauxensis*** A. Nováková & P.M. Martin-Sanchez, Fungal Biology 116 (5): 580 (2012)

Obligate synonyms: ***Scolecobasidium lascauxense*** (A. Nováková & P.M. Martin-Sanchez) G.Y. Sun & Lu Hao, Fungal Biology 12: 492 (2013)

***Eurotiomycetes***

***incertae sedis***

***Sarcinomyces*** Lindner, Mikroskopische Betriebskontrolle in den Gährungsgewerben: 228 (1898)

***Sarcinomyces sideticae*** Sert & Sterfl., Botanical Journal of the Linnean Society 154 (3): 379 (2007) Invalid

***Chaetothyriales***

***incertae sedis***

***Bacillicladium*** Hubka, Réblová, Thureborn, PLoS One 11 (10): 14 (2016)

***Bacillicladium lobatum*** Hubka, Réblová, Thureborn, PLoS One 11 (10): 17 (2016)

***Bradymyces*** Hubka, Réblová, Selbmann & M. Kolařík, Antonie van Leeuwenhoek 106 (5): 983 (2014)

***Bradymyces alpinus*** Hubka, Selbmann, Réblová & M. Kolařík, Antonie van Leeuwenhoek 106 (5): 985 (2014)

***Bradymyces graniticola*** Hubka, Réblová & Thureborn, PLoS One 11 (10): e0163396, 19 (2016)

***Bradymyces pullus*** L. Su, W. Sun & M.C. Xiang, Journal of Fungi 6 (4, no. 187): 15 (2020)

***Bradymyces yunnanensis*** L. Su, W. Sun & M.C. Xiang, Journal of Fungi 6 (4, no. 187): 16 (2020)

***Neophaeococcomyces*** *Crous & M.J. Wingf., Persoonia 35: 287 (2015)*

***Neophaeococcomyces catenatus*** (de Hoog & Herm.-Nijh.) Crous & M.J. Wingf., Persoonia 35: 287 (2015)

Basionym: ***Phaeococcus catenatus*** de Hoog & Herm.-Nijh., Studies in Mycology 15: 126 (1977)

Taxon synonyms: ***Phaeococcomyces catenatus*** (de Hoog & Herm.-Nijh.) de Hoog, Taxon 28: 348 (1979)

***Cyphellophoraceae***

***Cyphellophora*** G.A. de Vries, Mycopathologia et Mycologia Applicata 16: 47 (1962)

***Cyphellophora botryose***

***Cyphellophora guizhouensis***

***Herpotrichiellaceae***

***Cladophialophora*** Borelli, Proceedings of the 5th International Conference on Mycoses: 355 (1980)

***Cladophialophora humicola*** Crous & U. Braun, Studies in Mycology 58: 189 (2007)

***Cladophialophora nyingchiensis*** W. Sun, L. Su, M.C. Xiang & Xing Z. Liu, Journal of Fungi 6 (4, no. 187): 26 (2020)

***Cladophialophora tengchongensis*** W. Sun, L. Su, M.C. Xiang & X.Z. Liu, Journal of Fungi 6 (4, no. 187): 27 (2020)

***Cladophialophora tumbae*** Kiyuna, K.D. An, R. Kigawa & Sugiy., Mycoscience 59 (1): 80 (2017)

***Cladophialophora tumulicola*** Kiyuna, K.D. An, R. Kigawa & Sugiy., Mycoscience 59 (1): 81 (2017)

***Exophiala*** J.W. Carmich., Sabouraudia 5 (1): 122 (1966)

Taxon synonyms: ***Wangiella*** McGinnis, Mycotaxon 5 (1): 354 (1977)

Taxon synonyms: ***Foxia*** Castell., Journal of Tropical Medicine and Hygiene 11: 261 (1908) Invalid nomen nudum

***Exophiala angulospora*** Iwatsu, Udagawa & T. Takase, Mycotaxon 41 (2): 322 (1991)

***Exophiala bonariae*** Isola & Zucconi, Fungal Systematics and Evolution 3: 127 (2019)

Taxon synonyms: ***Exophiala bonariae*** Isola & Zucconi, Fungal Diversity 76: 85 (2015) Invalid Art. 40.7 (Shenzhen)

***Exophiala cinerea*** W. Sun, M.C. Xiang & Xing Z. Liu, Journal of Fungi 6 (4, no. 187): 28 (2020)

***Exophiala clavispora*** W. Sun, M.C. Xiang & Xing Z. Liu, Journal of Fungi 6 (4, no. 187): 29 (2020)

***Exophiala ellipsoidea*** W. Sun, L. Su, M.C. Xiang & X.Z. Liu, Journal of Fungi 6 (4, no. 187): 29 (2020)

***Exophiala nagquensis*** W. Sun, L. Su, M.C. Xiang & X.Z. Liu, Journal of Fungi 6 (4, no. 187): 30 (2020)

***Phaeococcomyces*** *de Hoog, Taxon 28: 348 (1979)*

***Phaeococcomyces nigricans*** (M.A. Rich & A.M. Stern) de Hoog, Taxon 28: 348 (1979)

Basionym: ***Cryptococcus nigricans*** M.A. Rich & A.M. Stern, Mycopathologia et Mycologia Applicata 9: 191 (1958)

Obligate synonyms: ***Phaeococcus nigricans*** (M.A. Rich & A.M. Stern) de Hoog, Studies in Mycology 15: 125 (1977)

Obligate synonyms: ***Melanocryptococcus nigricans*** (M.A. Rich & A.M. Stern) Della Torre & Cif.: 9 (1964)

Obligate synonyms: ***Nigrococcus nigricans*** (M.A. Rich & A.M. Stern) Novák & Zsolt, Acta Botanica Academiae Scientiarum Hungarica 7: 142 (1961)

***Phialophora*** Medlar, Mycologia 7 (4): 202 (1915)\

***Rhinocladiella*** Nannf., Svenska Skogsvårdsföreningens Tidskrift 32: 461 (1934)

Taxon synonyms: ***Racodium*** Pers., Neues Magazin für die Botanik 1: 123 (1794)

Taxon synonyms: ***Phialoconidiophora*** M. Moore & F.P. Almeida, Annals of the Missouri Botanical Garden 23: 548 (1936)

Taxon synonyms: ***Carrionia*** Bric.-Irag., Rev. Clin. Luiz Razetti, (Caracas): 121 (1938)

***Rhinocladiella atrovirens*** Nannf., Svenska Skogsvårdsföreningens Tidskrift 32: 462 (1934)

Taxon synonyms: ***Melanchlenus eumetabolus*** Calandron, Revue de Mycologie (Paris) 17: 190 (1953)

Taxon synonyms: ***Melanchlenus cumetabolus*** Calandron (1953)

***Trichomeriaceae***

***Anthracina*** L. Su, W. Sun & M.C. Xiang, Journal of Fungi 6 (4, no. 187): 12 (2020)

***Anthracina ramosa*** L. Su, W. Sun & M.C. Xiang, Journal of Fungi 6 (4, no. 187): 13 (2020)

***Anthracina saxicola*** L. Su, W. Sun & M.C. Xiang, Journal of Fungi 6 (4, no. 187): 14 (2020)

Obligate synonyms: ***Anthracina saxincola*** L. Su, W. Sun & M.C. Xiang (2020) Orthographic variant

***Lithohypha*** Selbmann & Isola, Fungal Diversity 86: 258 (2017)

Basionym: ***Lithophila*** Selbmann & Isola, Fungal Diversity 76: 88 (2015) Illegitimate Art. 40.1 (Melbourne); Art. 53.1, non Lithophila Sw. 1788 (Amaranthaceae)

Taxon synonyms: ***Lithophila*** Selbmann & Isola, Fungal Systematics and Evolution 3: 128 (2019) Illegitimate Art. 53.1, non Lithophila Sw. 1788 (Amaranthaceae)

***Lithohypha catenulata*** L. Su, W. Sun & M.C. Xiang, Journal of Fungi 6 (4, no. 187): 20 (2020)

***Lithohypha guttulata*** Selbmann & Isola, Fungal Diversity 86: 258 (2017)

Taxon synonyms: ***Lithophila guttulata*** Selbmann & Isola, Fungal Systematics and Evolution 3: 128 (2019) superfluous

Taxon synonyms: ***Lithophila guttulata*** Selbmann & Isola, Fungal Diversity 76: 90 (2015) invalid Art. 40.7 (Melbourne)

***Trichomerium*** Speg., Physis Revista de la Sociedad Argentina de Ciencias Naturales 4 (17): 284 (1918)

Taxon synonyms: ***Capnobatista*** Cif. & F.B. Leal ex Bat. & Cif., Saccardoa 2: 75 (1963)

Taxon synonyms: ***Triposporiopsis*** W. Yamam., Pap. Dedic. Tochinai & Fukushi Commem. 60th Birthdays: 52–56 (1955)

Taxon synonyms: ***Paropodia*** Cif. & Bat., Publicações do Instituto de Micologia da Universidade do Recife 36: 5 (1956)

***Trichomerium cicatricatum*** L. Su, W. Sun & M.C. Xiang, Journal of Fungi 6 (4, no. 187): 21 (2020)

***Trichomerium flexuosum*** W. Sun, X.Z. Liu & M.C. Xiang, Journal of Fungi 6 (4, no. 187): 23 (2020)

***Trichomerium lapideum*** L. Su, W. Sun & M.C. Xiang, Journal of Fungi 6 (4, no. 187): 24 (2020)

***Trichomerium leigongense*** W. Sun, L. Su & M.C. Xiang, Journal of Fungi 6 (4, no. 187): 25 (2020)

**Nontypical rock-inhabiting fungi**

***Ascomycota***

***Dothideomycetes***

***Cladosporiales***

***Cladosporiaceae***

***Cladosporium*** Link, Magazin der Gesellschaft Naturforschenden Freunde Berlin 7: 37 (1816)

Taxon synonyms: ***Heterosporium*** Klotzsch ex Cooke, Grevillea 5 (35): 122 (1877)

Taxon synonyms: ***Cladosporium*** subgen. Heterosporium (Klotzsch ex Cooke) J.C. David, Mycological Papers 172: 29 (1997)

Taxon synonyms: ***Beejadwaya*** Subram., Kavaka 5: 97 (1978)

Taxon synonyms: ***Acrosporella*** Riedl & Ershad, Sydowia 29 (1–6): 166 (1977)

Taxon synonyms: ***Azosma Corda***, Deutschlands Flora, Abt. III. Die Pilze Deutschlands 3 (12): 35 (1831)

Taxon synonyms: ***Mydonosporium*** Corda, Deutschl. Flora, III (Pilze): 95 (1833)

Taxon synonyms: ***Myxocladium*** Corda, Icones fungorum hucusque cognitorum 1: 12 (1837)

Taxon synonyms: ***Polyrhizium*** Giard, Bulletin Scientifique de la France et de la Belgique 20: 217 (1889)

Taxon synonyms: ***Spadicesporium*** V.N. Boriss. & Dvoïnos, Novosti Sistematiki Nizshikh Rastenii 19: 35 (1982)

Taxon synonyms: ***Sporocladium*** Chevall., Flore Générale des Environs de Paris 1: 35 (1826)

Taxon synonyms: ***Davidiella*** Crous & U. Braun, Mycological Progress 2 (1): 8 (2003)

***Pleosporomycetidae***

***Pleosporales***

***incertae sedis***

***Phoma*** Sacc., Michelia 2 (6): 4 (1880) [MB#9358]

Taxon synonyms

***Chlamydosporium*** Peyronel, I germi astmosferici dei fungi con micelio: 18 (1913)

***Leptophoma*** Höhn., Sitzungsberichte der Kaiserlichen Akademie der Wissenschaften Math.-naturw. Klasse Abt. I 124: 73 (1915)

***Macroplodiella*** Speg., Anales del Museo Nacional de Historia Natural Buenos Aires 17: 134 (1908)

***Paraphoma*** Morgan-Jones & J.F. White, Mycotaxon 18 (1): 58 (1983)

***Phomopsina*** Petr., Annales Mycologici 20: 145 (1922)

***Pseudosclerophoma*** Petr., Annales Mycologici 21 (3–4): 283 (1923)

***Rhizosphaerella*** Höhn., Hedwigia 59: 254 (1917) [MB#9729]

***Sclerophomina*** Höhn., Hedwigia 59: 240 (1917)

Vialina Curzi, Bolletino della Stazione di Patologia Vegetale di Roma 15: 252 (1935)

***Peyronellaea*** Goid., Atti della Accademia Nazionale dei Lincei Sér. 8, 1: 451 (1946)

***PericoniaceaePericonia*** Tode, Fungi Mecklenburgenses Selecti 2: 2 (1791)

Taxon synonyms

***Harpocephalum*** G.F. Atk., Bulletin of the Cornell University (Science) 3 (1): 41 (1897)

***Pachytrichum*** Syd., Annales Mycologici 23 (3–6): 420 (1925)

***Sporodum*** Corda, Icones fungorum hucusque cognitorum 1: 18 (1837)

***Trichocephalum*** Costantin, Revue agric. Sucr. Ile Maurice: 106 (1888)

***PleosporaceaeAlternaria*** Nees, System der Pilze und Schwämme: 72 (1817)

Taxon synonyms

***Embellisia*** E.G. Simmons, Mycologia 63: 380 (1971)

***Alternaria*** sect. Embellisia Woudenb. & Crous, Studies in Mycology 75: 190 (2013)

***Ulocladium*** Preuss, Linnaea 24: 111 (1851) [MB#10346]

***Alternaria*** sect. Ulocladium Woudenb. & Crous, Studies in Mycology 75: 205 (2013)

***Chmelia*** Svob.-Pol., Biológia Bratislava 21: 82 (1966) [MB#7626]

***Macrosporium*** Fr., Systema Mycologicum 3: 373 (1832) [MB#8821]

***Nimbya*** E.G. Simmons, Sydowia 41: 316 (1989) [MB#25376]

***Alternaria*** sect. Nimbya Woudenb. & Crous, Studies in Mycology 75: 197 (2013)

***Allewia*** E.G. Simmons, Mycotaxon 38: 260 (1990) [MB#25500]

***Lewia*** M.E. Barr & E.G. Simmons, Mycotaxon 25 (1): 289 (1986)

***Elosia*** Pers., Mycologia Europaea 1: 12 (1822)

***Prathoda*** Subram., Journal of the Indian Botanical Society 35 (1): 73 (1956)

***Rhopalidium*** Mont., Annales des Sciences Naturelles Botanique 6: 30 (1836)

***Trichoconiella*** B.L. Jain, Kavaka 3: 39 (1976)

***Crivellia*** Shoemaker & Inderbitzin, Canadian Journal of Botany 84 (8): 1308 (2006)

***Alternaria*** sect. Crivellia Woudenb. & Crous, Studies in Mycology 75: 189 (2013)

***Ybotromyces*** Rulamort, Bulletin de la Société Botanique du Centre-Ouest 17: 192 (1986)

***Chalastospora*** E.G. Simmons, CBS Biodiversity Series 6: 668 (2007)

***Alternaria*** sect. Chalastospora (E.G. Simmons) Woudenb. & Crous, Studies in Mycology 75: 188 (2013)

***Teretispora*** E.G. Simmons, CBS Biodiversity Series 6: 674 (2007)

***Alternaria*** sect. Teretispora Woudenb. & Crous, Studies in Mycology 75: 202 (2013)

***Botryomyces*** de Hoog & C. Rubio, Sabouraudia 20: 19 (1982)

***Brachycladium*** Corda, Icones fungorum hucusque cognitorum 2: 14 (1838)

***Sinomyces*** Yong Wang bis & X.G. Zhang, Fungal Biology 115 (2): 192 (2011)

***Undifilum*** B.M. Pryor, Creamer, Shoemaker, McLain-Romero & Hambl., Botany 87 (2): 190 (2009)

***Alternaria*** sect. Undifilum Woudenb. & Crous, Studies in Mycology 75: 206 (2013)

***Eurotiomycetes***

***Eurotiomycetidae***

***Eurotiales***

***Aspergillaceae***

***Aspergillus*** P. Micheli ex Haller, Historia stirpium indigenarum Helvetiae inchoata 3: 113 (1768)

Taxon synonyms

***Acmosporium*** Corda, Icones fungorum hucusque cognitorum 3: 11 (1839)

***Alliospora*** Pim, J. Bot., London: 234 (1883)

***Basidiella*** Cooke, Grevillea 6 (39): 118 (1878)

***Briarea*** Corda, Deutschlands Flora, Abt. III. Die Pilze Deutschlands 2–6: 11 (1831)

***Cladaspergillus*** Ritgen, Schr. Marb. Ges.: 89 (1831)

***Euaspergillus*** F. Ludw., Lehrbuch der Niederen Kryptogamen: 258 (1892)

***Gutturomyces*** Rivolta, Dei Parassiti Vegetali: 579 (1873)

***Raperia*** Subram. & Rajendran, Kavaka: 133 (1976)

***Rhodocephalus*** Corda, Icones fungorum hucusque cognitorum 1: 21 (1837)

***Rhopalocystis*** Grove, J. Econ. Biol.: 40 (1911)

***Sceptromyces*** Corda, Deutschlands Flora, Abt. III. Die Pilze Deutschlands 3 (11): 7 (1831)

***Aspergillus*** P. Micheli, Nova Plantarum Genera: 212, t. 92 (1729)

***Penicillium*** Link, Magazin der Gesellschaft Naturforschenden Freunde Berlin 3 (1): 16 (1809)

Taxon synonyms

***Torulomyces*** Delitsch, Ergebnisse der theoretischen und angewandten Mikrobiologie: Band I: Systematik der Schimmelpilze: 91 (1943)

***Penicillium*** sect. Torulomyces (Delitsch) Stolk & Samson, Advances in Penicillium and Aspergillus Systematics: 169 (1986)

***Floccaria*** Grev., Scott. crypt. fl.: pl. 301 (1827)

***Moniliger*** Letell. (1839)

***Pritzeliella*** Henn., Beiblatt zur Hedwigia 42: 88 (1903)

***Walzia*** Sorokin, Trudy Obshchestva ispytatelei prirody pri Imperatorskom Khar’kovskom universitê: 47 (1871)
